# Tissue-Based Mapping of the Fathead Minnow (*Pimephales promelas*) Transcriptome and Proteome

**DOI:** 10.3389/fendo.2018.00611

**Published:** 2018-11-06

**Authors:** Candice Lavelle, Ley Cody Smith, Joseph H. Bisesi, Fahong Yu, Cecilia Silva-Sanchez, David Moraga-Amador, Amanda N. Buerger, Natàlia Garcia-Reyero, Tara Sabo-Attwood, Nancy D. Denslow

**Affiliations:** ^1^Department of Environmental and Global Health, University of Florida, Gainesville, FL, United States; ^2^Center for Environmental and Human Toxicology, University of Florida, Gainesville, FL, United States; ^3^Department of Physiological Sciences, University of Florida, Gainesville, FL, United States; ^4^Interdisciplinary Center for Biotechnology Research, University of Florida, Gainesville, FL, United States; ^5^Environmental Laboratory, US Army Engineer Research & Development Center, Vicksburg, MS, United States

**Keywords:** fathead minnow, transcriptome, proteome, tissue-specific, endocrine system, proteogenomics

## Abstract

Omics approaches are broadly used to explore endocrine and toxicity-related pathways and functions. Nevertheless, there is still a significant gap in knowledge in terms of understanding the endocrine system and its numerous connections and intricate feedback loops, especially in non-model organisms. The fathead minnow (*Pimephales promelas*) is a widely used small fish model for aquatic toxicology and regulatory testing, particularly in North America. A draft genome has been published, but the amount of available genomic or transcriptomic information is still far behind that of other more broadly studied species, such as the zebrafish. Here, we used a proteogenomics approach to survey the tissue-specific proteome and transcriptome profiles in adult male fathead minnow. To do so, we generated a draft transcriptome using short and long sequencing reads from liver, testis, brain, heart, gill, head kidney, trunk kidney, and gastrointestinal tract. We identified 30,378 different putative transcripts overall, with the assembled contigs ranging in size from 264 to over 9,720 nts. Over 17,000 transcripts were >1,000 nts, suggesting a robust transcriptome that can be used to interpret RNA sequencing data in the future. We also performed RNA sequencing and proteomics analysis on four tissues, including the telencephalon, hypothalamus, liver, and gastrointestinal tract of male fish. Transcripts ranged from 0 to 600,000 copies per gene and a large portion were expressed in a tissue-specific manner. Specifically, the telencephalon and hypothalamus shared the most expressed genes, while the gastrointestinal tract and the liver were quite distinct. Using protein profiling techniques, we identified a total of 4,045 proteins in the four tissues investigated, and their tissue-specific expression pattern correlated with the transcripts at the pathway level. Similarly to the findings with the transcriptomic data, the hypothalamus and telencephalon had the highest degree of similarity in the proteins detected. The main purpose of this analysis was to generate tissue-specific omics data in order to support future aquatic ecotoxicogenomic and endocrine-related studies as well as to improve our understanding of the fathead minnow as an ecological model.

## Introduction

Omics technologies have significantly improved our understanding of how biological systems work. Their rapid development and the large amount of data generated allowed for the evolution of top-down approaches in order to understand systems that would complement the reductionist bottom-up approaches. These developments enabled rapid and broad characterization of many levels of biology through genome and transcriptome sequencing, proteomics, or metabolomics (1–4). Due to the extremely rapid advancement of sequencing technologies, it is now faster and more affordable than ever to generate data for genomics and transcriptomics analyses. As a result, omics techniques are increasingly being applied to “unusual” species to generate information that allows better understanding of novel biological characteristics (5, 6) in fields ranging from evolution and adaptation to toxicology and endocrine research (7). A key step in the development of omics applications for endocrine research is to refine their utilization in model species used in understanding both the highly conserved and the species-specific aspects of the endocrine system (6, 8). Here, we aim to further increase our knowledge of the fathead minnow to improve its usefulness as an ecological and endocrine model.

The fathead minnow (FHM, *Pimephales promelas*) is a member of the Cyprinidae family with a broad distribution in aquatic environments, both in running and still waters, across North America (9). They tolerate a wide range of water characteristics, including pH, alkalinity, and temperature (9–11). Fathead minnows are sexually dimorphic and have a rapid life cycle, with a well-defined developmental process, reproductive cycle, and behavior (12–15). All of these characteristics together with the well-established methods for its culture and husbandry (16) make the FHM suitable as an ecologically relevant fish model. In fact, the FHM is the most frequently used small fish model for regulatory ecotoxicology in North America since the 1950s (17). After the US Environmental Protection Agency was established in 1970, the FHM was adopted as a primary model organism for standardized regulatory toxicity testing, leading to the development of numerous testing guidelines (18–20). As a consequence, the extensive toxicity data available offers the FHM the greatest potential for linking molecular diagnostic indicators to ecologically relevant outcomes (17).

The relatively recent interest in contaminants that act as endocrine disruptors has focused on effects on the endocrine system of fish, since these organisms are present in contaminated environments. Studies analyzing effects on reproduction (21–23), thyroid function (24, 25), neuroendocrine control (26–28) or effects on sex differentiation during sensitive periods of development (29–32) require good molecular tools for data interpretation. Thus it is important to develop well-annoted sequence databases to have a more comprehensive evaluation of the effects of endocrine disruptors on fathead minnows using functional genomic approaches. In addition, it is important to understand the physiology and endocrinology of this useful species. However, significantly less genetic information is available for the FHM than other models such as the zebrafish (*Danio rerio*), which has an assembled reference genome (https://www.ncbi.nlm.nih.gov/grc/zebrafish).

The first FHM draft genome was published in 2016 (33) and was produced from Illumina sequencing at 120X coverage. The genome annotation was later improved, leading to a total of 43,345 gene predictions (34). In addition, a web-accessible genome browser was created, which enables simplified access to the sequence data and its associated annotations (https://www.setac.org/page/fhmgenome). Nonetheless, it is crucial to continue increasing our basic understanding of the FHM model by expanding on genome annotation studies, including characterizing both the transcriptome and proteome. This will further facilitate its use in a broad range of applications: from endocrine-related studies, to predictive toxicology and development of computational models, and its use as a surrogate to study other species, including those that are threatened and endangered.

The main objective of this study was to increase the value of the FHM as a model by creating comprehensive transcriptomic and proteomic databases. This study also aims to survey tissue-specific baseline transcriptomic and proteomic expression profiles in select endocrine active organs in adult male FHM to support aquatic ecotoxicogenomic studies.

## Materials and methods

### Fish rearing

All fish husbandry was conducted under the supervision of the University of Florida Institutional Animal Care and Use Committee. Adult fathead minnows (*Pimephales promelas*) were obtained from an in-house culture at the Aquatic Toxicology Core Laboratory at the University. Fish were maintained in the laboratory in flow-through systems of dechlorinated tap water prior to selection for sequencing experiments.

Fish were sacrificed at different times for three different experiments by submersion in buffered 250 mg/L MS-222 (Western Chemical). Fish tissues were harvested for each experiment and flash-frozen in liquid nitrogen and stored at −80°C until needed. For the PacBio experiment, tissues were harvested from a single male fish, including the whole brain, gut, liver, gonad, heart, gill, head kidney, and trunk kidney. For the RNA-seq experiment, three individual male fish were used, and tissues collected included the telencephalon, hypothalamus, liver, and gut, and the same 4 tissues were collected from two male fish for the proteomics experiment.

### RNA extraction and sequencing

Tissue extractions followed procedures previously described (35, 36). Briefly, tissues were homogenized in RNA Stat-60 (TelTest) using a handheld rotary homogenizer followed by organic separation with chloroform. RNA was then subjected to a second round of RNA Stat-60/Chloroform extraction, followed by precipitation in isopropanol overnight at −20°C. RNA was washed twice with 75% ethanol, dried, and reconstituted in RNAsecure (ThermoFisher Scientific). Reconstituted RNA was DNase-treated to remove possible genomic DNA contamination using Turbo DNase (ThermoFisher Scientific). The quality of the RNA was assessed using an Agilent Bioanalyzer 2100. Only samples with RNA integrity numbers (RINs) exceeding 8 were used for sequencing. Samples were then quantified using a ThermoFisher Scientific Qubit 3.0 fluorimeter.

For the PacBio sequencing, an RNA pool was created by adding equal mass of RNA from each of the extracted tissues (brain, liver, gut, testes, heart, gill, head kidney, and trunk kidney) into the pool. Pools were delivered to the Interdisciplinary Center for Biotechnology Research (ICBR) Sequencing Core Laboratory. For the RNA-seq experiments telencephalon, hypothalamus, liver, and gut tissues from three different fish were kept separate for downstream analysis.

For RNAseq, library preparation and sequencing were performed by Global Biologics LLC (Columbia, MO, USA). Total RNA was quantitated using a Qubit RNA assay kit and Qubit 2.0 fluorometer (Life Technologies Inc.), and RNA integrity was confirmed using the standard sensitivity Fragment Analyzer Total RNA Assay and System (Advanced Analytical Inc.). Briefly, five hundred nanograms of total RNA was used as input material for the Illumina TruSeq Directional v2 high-throughput library construction procedure (Illumina Inc.). Messenger RNA was enriched from total RNA using oligo-dT magnetic beads and fragmented to ~100–300 bp with a single shearing and RT primer hybridization step before generating first- and second-strand cDNA. The resulting DNA was prepared for sequencing by blunt end repair, 3′ adenylation, multiplex compatible adapter ligation (containing TruSeq indexes), and PCR amplification (98°C for 30 s, 11–13 cycles [98°C for 10 s, 60°C for 30 s, and 72°C for 30 s], 72°C for 5 min, and 10°C hold). Library validation was performed using the Fragment Analyzer (Advanced Analytical Inc.) followed by quantitation using the Qubit HS DNA Assay and qPCR Kit for Illumina (Kapa Biosystems Inc). Libraries were diluted based on the quantitation obtained using the Qubit fluorometer and sequenced using one lane (paired-read 100 bp sequencing) on the HiSeq 4000 platform (Illumina Inc.).

### Long read sequencing for transcriptome construction

Long read sequencing was performed using the Pacific Biosystems RSII long read sequencer. Full-length, RNA sequencing libraries (i.e., Iso-Seq^TM^) were constructed according to the recommended protocol by PacBio (37, 38), with a few modifications. Briefly, only RNA preparations with a RIN ≥ 8 were used, as indicated by the Agilent BioAnalyzer or TapeStation. RNA preparations of similar quality from brain, liver, gut, testes, heart, gill, head kidney, and trunk kidney from one male fathead minnow were pooled and used for IsoSeq as a single sample. Briefly, one microgram of total RNA from the pool described above was converted to full-length cDNA using the SMRTer PCR cDNA synthesis reagents (Cat. # 634925) (Clontech, Palo Alto, CA). The number of cDNA amplification cycles was optimized to generate sufficient material that could be used for PacBio SMRT bell library construction over four fraction sizes (0.8–2 kb, 2–3 kb, 3–5 kb, and >5 kb). Fourteen amplification cycles were required. Full-length total cDNA was placed on the ELF SageSciences system (Electrophoretic Lateral Fractionation System). Twelve cDNA fractions were collected, of varying size between 0.8 and ~15 kb. Further amplification was needed to generate enough material (for library construction) for the two larger size bins. Additional amplification of the larger size bins resulted in small size byproducts. Therefore, a second size selection (for 3–5 and >5 kb fragments) was performed using an 11 cm x 14 cm agarose slab gel. Library-polymerase binding was done at 0.01–0.04 nM (depending on library insert size) for sequencing on the PacBio RSII instrument. Diffusion loading was used for the short fragments, while MagBead loading was used for the larger fragments.

Sample cleaning of SageELF fractions and SMRT bell library construction was done following the manufacturer's protocols (39). In brief, fractions were purified using AMPure magnetic beads (0.6:1.0 beads to sample ratio). Final libraries were eluted in 15 μL of 10 mM Tris HCl, pH 8.0. Library fragment size was estimated by the Agilent TapeStation (genomic DNA tapes), and this data was used for calculating molar concentrations. Between 75 and 125 pM of library from each size fraction was loaded onto eight SMRT cells for PacBio RS II sequencing. All other sequencing steps were done according to the recommended protocol by the PacBio sequencing calculator and the *RS Remote Online Help* system.

### Bioinformatics

#### *De novo* assembly

The raw reads generated from multiple insert-size libraries by PacBio RSII sequencer were processed with PacBio SMRT portal system. The ROI (reads of inserts) from subreads, including the full-length non-chimeric reads, were produced by RS_IsoSeq (40). The iterative clustering for error correction (ICE) algorithm and Quiver were applied for improving isoform accuracy and removing redundancy (Table 1). All isoform sequences were further clustered and assembled with PTA version 3.0.0 (Paracel Transcript Assembler) (Paracel Inc, Pasadena, CA).

**Table 1:** PACBio sequencing data.

**Libraries**	**SMRT cells**	**ROI**	**Full length of ROI**	**Mean length of ROI**	**Mean quality of ROI**	**Mean passes**
0.8–2kb	2	96194	61014	1133	0.95	17
2–3kb	2	92862	37347	1736	0.89	6.6
3–5kb	2	104117	16308	2216	0.86	2.5
>5kb	2	71778	14094	2997	0.88	4.5
Total	8	364951	128763	2020.5	0.895	

Raw sequencing data generated from illumina NextSeq 500 system were processed with the program Cutadapt (41) to trim off sequencing adaptors, primers, and low-quality bases with respect to a quality value cutoff of 20 (phred-like score). With masking and trimming sequencing repeats, primers and/or adaptors used in cDNA library preparation and normalization, the resulting reads with > = 40 bp were assembled using Trinity (42), SOAPdenovo (43), and Newbler assembler (version 2.8). A hybridized transcriptome assembly of the contigs with ≥ 75 bp from Trinity, SOAPdenovo, and Newbler was performed with PTA version 3.0.0 (Paracel Transcript Assembler) (Paracel Inc, Pasadena, CA). In PTA, the low-quality bases were trimmed and the sequences with length <75 bp and the mitochondrial and ribosomal RNA genes of FHM were excluded from consideration during initial pair-wise comparison. After cleanup, sequences were passed to the PTA clustering module for pair-wise comparison and then to CAP3-based PTA assembly module for assembly.

The consensus sequences resulting from the PTA were annotated against the NCBI NR and NT databases. For each query sequence, the top 100 blast hits were retrieved and the best scoring hit and the tentative GO term from Gene Ontology with e-value ≤ 1e-4 were annotated to query sequences. These GO term assignments were organized around GO hierarchies divided into biological processes, cellular components, and molecular functions. In addition, we also characterized the assembled sequences with respect to functionally annotated genes by BLAST searching against the NCBI reference sequences (RefSeq) of *Danio rerio* (46,757 transcripts).

#### Analysis of RNA-seq data

Reads acquired from the illumina HiSeq 4000 sequencing platform were cleaned up with the Cutadapt program to trim off sequencing adaptors and low-quality bases with a quality phred-like score < 20. Reads < 40 bases were excluded from RNA-seq analysis. The transcriptome consensus sequences were used as reference sequences for RNA-seq analysis. The cleaned reads of each sample were mapped independently to the *Danio rerio* reference sequences using the mapper of bowtie 2 with a maximum of 3 mismatches for each read. The mapping results were processed with samtools and scripts developed in house at ICBR to remove potential PCR duplicates and choose uniquely mapped reads for gene expression analysis.

Differential gene expression was determined as follows: The number of mapped reads for each individual gene was counted using scripts developed in house at ICBR and analyzed by the DEB application for all pairwise comparisons using the edgeR algorithm and a 5% FDR cutoff (44). Significant up- and down-regulated genes were selected using the FDR adjusted *p*-value, fold-change, or both for downstream analysis.

### Confirmation of RNAseq transcripts with quantitative PCR

To cofirm the expression of select transcripts from the RNAseq data set, five healthy male fathead minnows were obtained from culture at the Center for Environmental and Human Toxicology, euthanized, and hypothalamus, telencephalon, liver and gut tissues were collected for RNA extraction and analysis. RNA extraction followed the same procedures described above for RNAseq. Primers were designed and validated for the following transcripts: lipoprotein lipase (lpl), estrogen receptor βb (*er*β*b*), peptide transporter 1 (*pept1*), and cytochrome P450 19a1b (*cyp19a1b*). Primer Sequences and conditions are found in Supplementary Table 1. Isolated RNA was reverse transcribed into cDNA (Quanta cDNA synthesis kit), and mixed with forward and reverse primers and SYBR Green for amplification and measurement on the BioRAD CFX96 Real-Time PCR Detection System using the following cycling parameters: 95°C for 3 min followed by 40 cycles of 95°C for 10 s, 58–60°C for 30 s (see Supplementary Table 1 for gene specific annealing temperatures). Replicate gene expression Cq values were normalized to the average Cq value for the hypothalamus for each gene, and presented as average fold change ± standard deviation in each tissue compared to the hypothalamus.

### Protein extraction and digestion

Tissue samples were mechanically disrupted in 300 μL RIPA buffer (25 mM Tris–HCl, pH 7.6, 150 mM NaCl, 1% nonylphenoxylpolyethoxylethanol-40, 1% sodium deoxycholate and 0.1% SDS) (Thermo) containing a protease inhibitor tablet (proprietary formulation containing AEBSF HCl, aprotinin, bestatin, E-64, leupeptin, pepstatin, EDTA) (Pierce) and subsequently incubated on ice for 30 min with intermittent vortexing. Samples were spun at 10,000 x g for 20 min at 4°C and supernatants were removed and protein content quantified by Bradford Protein Assay (Biorad). To 100 μL of supernatant, 400 μL of methanol was added followed by vigorous vortexing. Chloroform was added at 1:4 v/v methanol and samples were vigorously vortexed. Thereafter, 300 μL ddH_2_O was added to the samples and vigorously vortexed. Samples were then spun at 14,000 x g for 2 min at room temperature, the top aqueous layer was removed, and 400 μL methanol was added followed by vigorous vortexing. Samples were spun at 14,000 x g for 3 min and methanol was removed. Samples were dried and resuspended in 100 μL RIPA buffer containing protease inhibitor tablets.

Total protein (100 μg) from each sample was acetone-precipitated. The samples were dissolved in 0.1% SDS, 0.5 M triethylammonium bicarbonate (TEAB), pH 8.5; then reduced, alkylated, trypsin- (Promega, USA) digested and labeled according to manufacturer's instructions (ABsciex Inc. USA). Extra labels were quenched by adding 100 μL of ultrapure water and left at room temperature for 30 min. After quenching, samples were mixed together and dried down in a speedvac. The peptide mixtures were cleaned up with C18 spin columns according to manufacturer's instructions (Supelco, USA). Sample labeling was as follows; gut tissue biological replicates (113 and 118), hypothalamus biological replicates (114 and 117), telencephalon biological replicates (115 and 119), and liver biological replicates (116 and 121). The samples were then dissolved in strong cation exchange (SCX) solvent (25% v/v ACN, 10 mM ammonium formate, pH 2.8) and injected onto a Agilent HPLC 1100 system using a polysulfoethyl A column (2.1 mm x 100 mm, 5 μm, 300 Å, PolyLC, Columbia, USA). The peptides were eluted at a flow rate of 200 μL/min with a linear gradient from 0 to 20% solvent B (25% v/v ACN, 500 mM ammonium formate) over 80 min, followed by a ramping up to 100% solvent B in 5 min and holding for 10 min. The peptides were detected at 214 nm absorbance and a total of 10 fractions were collected.

### Mass spectrometry

Each SCX fraction was lyophilized in a speedvac and resuspended in loading buffer (3% acetonitrile, 0.1% acetic acid, 0.01% TFA) and cleaned up with C18 ZipTips according to manufacturer's instructions (Ziptip Millipore). After C18 solid phase extraction, samples were resuspended in loading buffer and 10 μL was injected onto an Acclaim Pepmap 100 precolumn (20 mm x 75 μm; 3 μm-C18) and then separated on a PepMap RSLC analytical column (250 mm x 75 μm; 2 μm-C18) at a flow rate of 350 nL/min on a 1200 nano Easy LC (Thermo Fisher). Solvent A composition was 0.1% formic acid (v/v); whereas solvent B was 99.9% ACN v/v, 0.1% formic acid (v/v). Peptide separation was performed with a linear gradient from 2 to 24% solvent B for 95 min, followed by an increase to 98% solvent B over 15 min and final hold for 10 min. Eluted peptides were directly sprayed onto an Q Exactive Plus hybrid quadrupole-Orbitrap mass spectrometer (ThermoFisher Scientific) for MS/MS analysis. The instrument was run on a data-dependent mode with a full MS scan 400–2,000 m/z and resolution of 70,000. MS/MS experiments were performed for the top 10 most intense ions using the following settings: an HCD NCE = 28%, isolation width = 3 Th, first mass = 105 Th, 5% underfill ratio, peptide match set to “preferred,” and an AGC target of 1e6. Dynamic exclusion for 60 s was used to prevent repeated analysis of the same peptides. The mass spectrometry proteomics data have been deposited to the ProteomeXchange Consortium via the PRIDE (45) partner repository with the dataset identifier PXD010216. An excel spreadsheet containing mass spectra information for identifying the proteins is found in supplementary information.

### Database searching and protein identification

A custom database was constructed for searching protein identification. This database was a composite of an in-house FHM protein database and the zebrafish (*Danio rerio)* database on uniprot. The in-house FHM database was created by selecting the longest open reading frame from the 6-frame translation of each sequence in our transcriptome database consisting of the PacBio reads generated in this study and reads from previous sequencing data from our labs in Blast2Go with the ORF Predictor function. The software chose the longest open reading frame for each sequence, which was subsequently annotated against zebrafish NR database using blastx and blastp and resulted in 56,099 annotated sequences. Once combined with the Uniprot zebrafish protein database our composite database consisted of 117,445 sequences.

The identification and quantification of proteins were performed using ProteinPilot™ Software 5.0.1 (AB SCIEX, Concord, ON) utilizing the Paragon and Progroup algorithms. The previously described protein database was appended before use to include common lab contaminants, and then the entire search field was doubled by the inclusion of decoys for calculating the FDR by the target-decoy method. The search parameters were as follows: iTRAQ 8-plex (peptide labeled), MMTS as a fixed modification on cysteine, trypsin digestion, orbi MS (1-3 ppm), Orbi MS/MS, no special factors, and ID focus of biological modifications and amino acid substitutions. The Unused ProtScore (Conf) was set at > 0.05 (10.0%) and *p*-value < 0.05 to ensure that quantitation was based on at least three unique peptides.

Additionally, because iTRAQ is a relative quantitation method, all data are reported as ratios of expression against another tissue, we chose hypothalamus. Our samples were expected to have a high percentage of differentially expressed proteins because they originate from different tissues; therefore, no bias or background corrections were applied. For a protein to be used for quantitative analysis and downstream pathway analysis it had to meet a series of conditions: it had to be identified at a 1% global FDR and ratio calculation *p*-value of < 0.05. Quantified proteins with a *p*-value >0.05 were not supported with enough evidence to reject the null hypothesis that differences observed in iTRAQ label ratios were random. For each replicate, the ratio to both normalizing hypothalamus replicates was averaged in log space. Then both replicates for each tissue were averaged in log space to calculate the overall tissue ratio.

### Pathway analysis

Subnetwork enrichment analysis (SNEA) was conducted in PathwayStudio^TM^ 10 (Elsevier) operating on the ResNet 11.0 mammalian database using the Fisher's Exact Test Subnetwork Enrichment Analysis option limiting subnetworks to those with *p* < 0.05.

## Results and discussion

**Figure 1:**
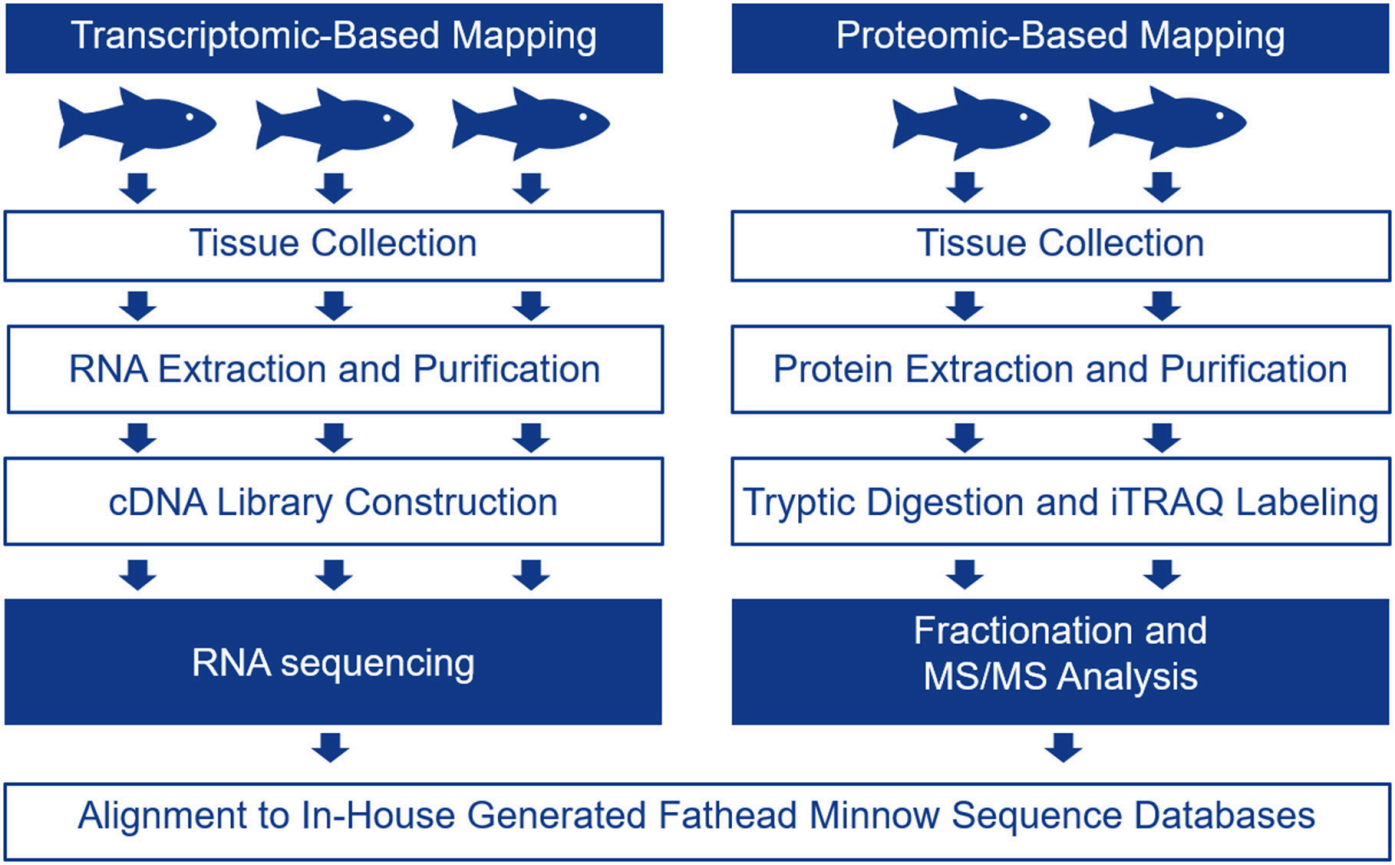
Schematic showing the experimental protocol followed to generate tissue-specific mRNA and protein data for four tissues including the telencephalon, hypothalamus, liver, and gut. Tissue-specific mRNA expression was evaluated with RNA-seq using 3 male biological replicates while tissue-specific protein expression was evaluated using iTRAQ in 2 male biological replicates.

The FHM is the model of choice for ecotoxicology in North America as there are many studies relating toxicant exposures to changes in apical endpoints in these fish [for a review, please see (17)]. In the present study, we chose one male FHM for single DNA molecule sequencing using the PacBio instrument in order to generate long reads. The transcriptome for FHM was assembled and it was used as a scaffold for interpreting RNA-seq and proteomics data to determine tissue-specific transcripts. The schematic in Figure 1 describes the overall experimental approach.

### Generation of FHM transcriptome

To generate a good quality transcriptome for FHM, we utilized the PacBio instrument, which provides single molecule, full-length transcript sequencing. This instrument can sequence very long reads (up to 100 kb) directly from a single DNA molecule (46). This technology sequences DNA from a closed circle using a template called the SMRTbell, which can diffuse into a nano-well called the zero-mode waveguide [for more information about the technology, please see (47)]. The circles can be very large and encompass an entire mRNA. This is the ideal instrument to assemble a transcriptome and aid the assembly of a reference genome. One disadvantage that has been pointed out by several studies is its relatively high error rate, about 11–15%, on any read. However, it is possible to work around this error rate as the errors are distributed randomly and the machine can read around the circle multiple times. It has been estimated that a 99% sequence fidelity can be determined by lining up the multiple sequences. PacBio reads are typically longer than the full-length cDNA sequence, allowing each molecule to go through several passes of sequencing. This routinely works, as the read length is up to 100 kb (47).

**Figure 2:**
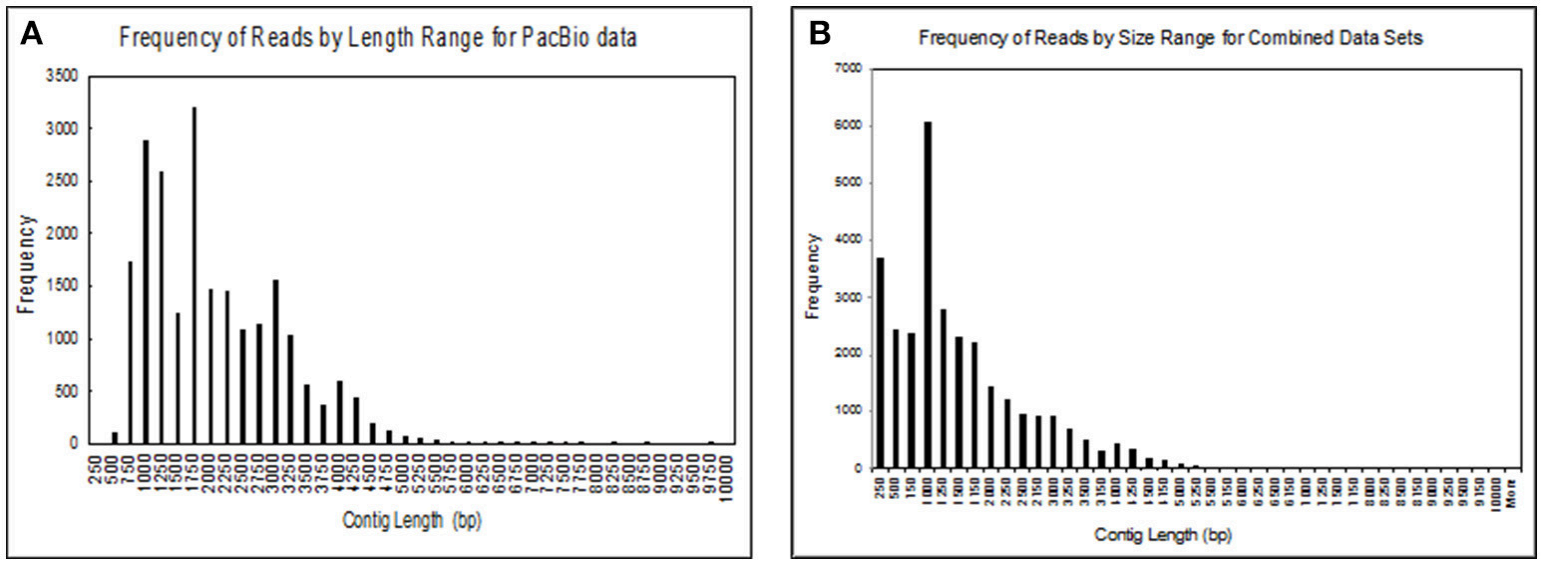
Size distribution of contigs obtained from **(A)** sequences obtained from the Pac Bio instrument for brain, liver, gut, testes, heart, gill, head kidney, and trunk kidney from one male fathead minnow; **(B)** assembled sequences from the PacBio data with several Illumina RNAseq datasets including the one performed in this study. The assembled sequences include transcripts from male and female fathead minnows obtained in various other experiments.

We obtained 30,385 reads from PacBio sequencing, covering a large portion of the transcriptome for a single male. The read lengths ranged from 264 to over 9,720 nts. We binned the sequences into groups based on their lengths with 250 nts per group, giving us 40 different groups (Figure 2A). We had 17,382 transcripts that were ≥1,000 nts and 182 that were ≥5,000 nts. At the high end of the distribution the five longest transcripts ranged from 7,726 to 9,720 nts long. In addition to transcripts identified by PacBio sequencing, we added sequences that we obtained from several Illumina RNA-seq projects for a large group of fathead minnows. This addition greatly increased the coverage of shorter contig lengths and enhanced some of the longer sequences giving us 21,183 transcripts >1,000 nts and 308 transcripts >5,000 nts (Figure 2B).

In preparing libraries of cDNA for sequencing by the PacBio instrument, it is possible to use barcodes to identify sequences from different tissues. However, in the present investigation, due to cost, we decided to pool RNAs from a variety of tissues and used a strategy that would ensure some long reads. Also, we wanted to enhance sequences that may lead to the identification of splice variants, as the PacBio is the ideal Next Gen sequencer for this purpose (48). For this work, we used a single adult male FHM, to prevent confounding by single polymorphic sequences from a population of fish (Manuscript in preparation).

### Tissue-specific transcriptome information for FHM

**Figure 3:**
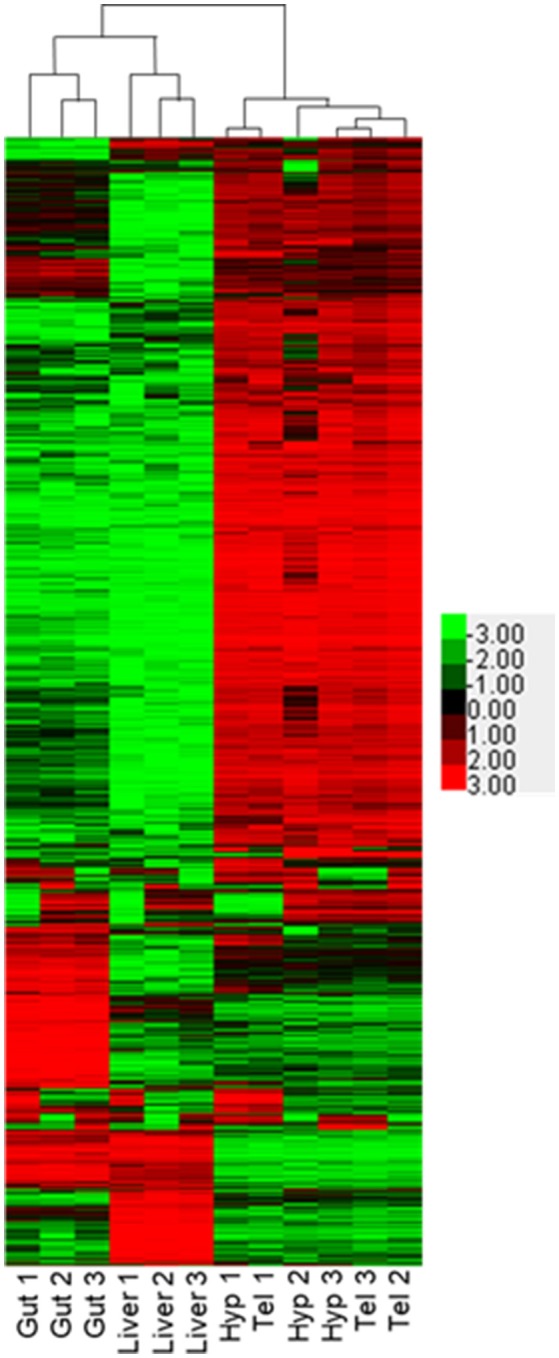
Comparison of transcriptional profiles for gut, liver, hypothalamus, and telencephalon. Heat map depicts Log2 (FPKM) of transcripts identified with at least 50 counts in at least three samples and a Max Value-Min Value >5. Hierarchical clustering was performed using Cluster 3.0 and visualized using Java Treeview.

We performed RNA-seq on hypothalamus, telencephalon, liver and gut of three different adult male FHMs to evaluate tissue-specific expression of genes. For a review of RNA-seq methodologies, please see Bayega et al. (49). As expected, each of the tissues, composed of different cell types, showed specific expression fingerprints. Overall, the RNA aligned to 30,378 different putative transcripts in our database. Transcript copies ranged from 0 to 600,000 copies. The mean number of copies of mRNAs in our sampling per tissue ranged from 80 to 266 when sequences with >50 hits were excluded. This is an arbitrary cut off, as some genes with important cellular functions may be expressed with lower copy number, but we think it is a reasonable cut off as estrogen receptor 2b (esr2b) ranged from 243 counts in the telencephalon to 2,517 counts in the liver, values similar to those published by Filby and Tyler using real time qPCR in adult male fathead minnows (50) Similarly, esr2a ranged from 35 in the telencephalon to 195 in the gut, relative values again similar to published data. Additionally, there were very low number of hits in males for esr1. Published data indicates that esr1 should be high in the liver of males and not found in the other tissues (50) and while we also found that to be the case in our study, the number of hits were well below our cutoff of 50 hits per gene. Pairwise comparisons were made for each tissue for all transcripts that were measured in at least 2 replicates of at least 1 tissue (Supplementary Figure 1). Overall, 28,616 transcripts met the requirements for statistical testing in DEB. Of those, 12,610 transcripts were not changed in any of the tissues. These are likely important housekeeping genes that are essential for all tissues. The number of significantly different transcripts varied by tissue and were 200 for hypothalamus to telencephalon (Supplementary Figure 1A), 11,282 transcripts comparing the hypothalamus to liver (Supplementary Figure 1B), 10,775 transcripts comparing the liver to telencephalon (Supplementary Figure 1C), 10,816 for the gut to telencephalon (Supplementary Figure 1D), 6,237 for gut to hypothalamus (Supplementary Figure 1E), and 10,143 for gut to liver (Supplementary Figure 1F). Comparison of expressed genes in the four tissues analyzed is shown in Figure 3. It is clear from this heatmap that the telencephalon and hypothalamus share the most expressed genes, with the three biological samples intermingling in the figure, while the gut and the liver are quite distinct. A recently published study mapping the human proteome also found lower correlations between brain and digestive tissues and higher correlations between liver and digestive tissues when investigating transcript expression (4).

A better and more holistic approach to analyzing the data is to compare subnetworks of genes involved in cellular processes for each of the tissues (Tables 2–**5**, Supplementary Tables 2–5). To do this, FHM transcripts were converted to human homologs, and transcripts that shared the same human homolog were summed. Transcript counts were normalized to the hypothalamus to compare to the proteomics data. Transcripts that were expressed at least 2-fold higher than in the hypothalamus were imported into PathwayStudio^TM^ for SNEA.

**Table 2:** Subnetwork enrichment analysis of gene sets specific for the gastrointestinal tract.

**#**	**Total # of neighbors**	**# of measured neighbors**	**Gene set seed**	**Median change**	***p*-value**
1	215	50	Intestinal absorption	15.92	2.85E-07
2	122	17	Gut development	31.84	1.19E-05
3	72	18	Lipid absorption	47.80	5.68E-05
4	139	27	Lipid export	19.38	2.99E-04
5	102	19	Bile secretion	29.33	3.77E-04
6	155	18	Lipoprotein metabolism	15.17	8.50E-04
7	44	10	Gastrointestinal system absorption	106.84	8.68E-04
8	66	16	Drug transport	29.33	1.33E-03
9	47	9	Gastrointestinal system digestion	115.65	1.45E-03
10	573	47	Energy homeostasis	6.83	1.71E-03
11	209	19	Transcytosis	8.63	1.85E-03
12	100	15	Intestine function	29.33	1.96E-03
13	245	22	Fluid secretion	7.26	2.00E-03
14	159	22	Intestine barrier	23.44	2.13E-03
15	55	13	Gallstone formation	31.43	2.68E-03

As expected, SNEA revealed tissue-specific enrichment of cellular processes relevant to known functions of each tissue. For example, 76 cellular processes had a *p*-value < 0.05 in the gut, including intestinal absorption, gut development, lipid absorption, gastrointestinal system absorption, and gastrointestinal system digestion (Table 2). In the liver, 48 cellular processes had *p*-values < 0.05 including fibrinolysis, liver development, hepatic regeneration, glycogenesis and glycogen degradation, and liver metabolism (Table 3). For the hypothalamus, 100 cellular processes had *p*-values < 0.05 and are involved in a myriad of processes such as neuron and brain development, nervous system development, neurogenesis, axon cargo transport, locomotion, neuroimmunomodulation, pituitary gland function and hormone generation, transmission of nerve impulse and nerve regeneration and potential, and neuroprotection and neurotransmitter uptake (Table 4), underscoring the importance of this part of the brain in controlling multiple organs and their functions. Finally, only 16 cellular processes in the telencephalon had *p*-values < 0.05 (Table 5) and included neuron development, neurogenesis, axogenesis, stem cell proliferation, neuron differentiation, and neuronal plasticity. As expected, there was a lot of overlap between the hypothalamus and the telencephalon, but discrete differences could also be identified.

**Table 3:** Selected subnetwork enrichment pathways for the liver.

**#**	**Total # of neighbors**	**# of measured neighbors**	**Gene set seed**	**Median change**	***p*-value**
1	174	18	Fibrinolysis	81.23	6.46E-06
2	78	15	Blood clot lysis	81.23	7.66E-06
3	242	13	Neutrophil chemotaxis	215.03	3.36E-04
6	158	16	microcirculation	25.18	3.01E-03
8	81	8	Sex maturation	90.17	3.17E-03
13	330	26	Liver development	17.51	1.13E-02
21	438	40	Hepatic regeneration	9.67	1.66E-02
22	409	23	Tissue remodeling	13.26	1.72E-02
30	325	12	Immunomodulation	47.50	2.81E-02
31	631	41	Fertilization	8.51	3.11E-02
33	129	11	Leukocyte accumulation	10.00	3.41E-02
34	52	8	Glycogenesis	70.15	3.45E-02
37	106	10	Glycogen degradation	9.45	3.73E-02
44	228	31	Liver metabolism	7.90	4.64E-02
46	72	11	Lipid absorption	7.90	4.73E-02

**Table 4:** Selected subnetwork enrichment pathways for the hypothalamus.

**#**	**Total # of neighbors**	**Overlap**	**Percent overlap**	**Gene set seed**	***p*-value**
4	319	7	2	Neuron development	6.03E-05
5	1,100	12	1	Brain development	1.31E-04
9	1,017	11	1	Nervous system development	2.78E-04
12	1,405	13	0	Neurogenesis	3.36E-04
15	338	6	1	Axon cargo transport	6.46E-04
16	951	10	1	Locomotion	6.76E-04
30	16	2	11	Neuroimmunomodulation	1.26E-03
32	159	4	2	Pituitary gland function	1.56E-03
35	887	9	1	Transmission of nerve impulse	1.64E-03
36	19	2	10	Olfactory bulb development	1.75E-03
37	430	6	1	Nerve regeneration	2.21E-03
52	106	3	2	Nerve potential	4.53E-03
87	944	8	0	Neuroprotection	8.77E-03
94	49	2	4	Neurotransmitter uptake	1.06E-02
95	50	2	3	Hormone biosynthesis	1.10E-02

**Table 5:** Selected subnetwork enrichment pathways for the telencephalon.

**#**	**Total # of neighbors**	**# of measured neighbors**	**Gene set seed**	**Median change**	***p*-value**
1	264	10	Neuron development	9.19	2.23E-03
2	105	7	Forebrain development	6.26	3.31E-03
3	1,129	21	Neurogenesis	3.31	5.76E-03
4	182	7	Cell fate specification	3.80	2.08E-02
5	425	9	Axonogenesis	4.01	2.23E-02
6	2,002	26	Transcription activation	3.31	2.36E-02
7	492	9	Stem cell proliferation	2.37	3.51E-02
8	136	5	Neurulation	6.26	3.57E-02
9	478	6	Organogenesis	4.30	3.68E-02
10	471	8	Neuronal migration	5.87	4.04E-02
11	5,848	63	Cell differentiation	2.77	4.17E-02
12	346	7	Axon guidance	3.38	4.23E-02
13	207	10	Neuron differentiation	2.82	4.34E-02
14	6,886	62	Cell proliferation	2.61	4.83E-02
15	1,107	23	Cell fate	2.42	4.86E-02
16	446	6	Neuronal plasticity	7.95	4.89E-02

**Figure 4:**
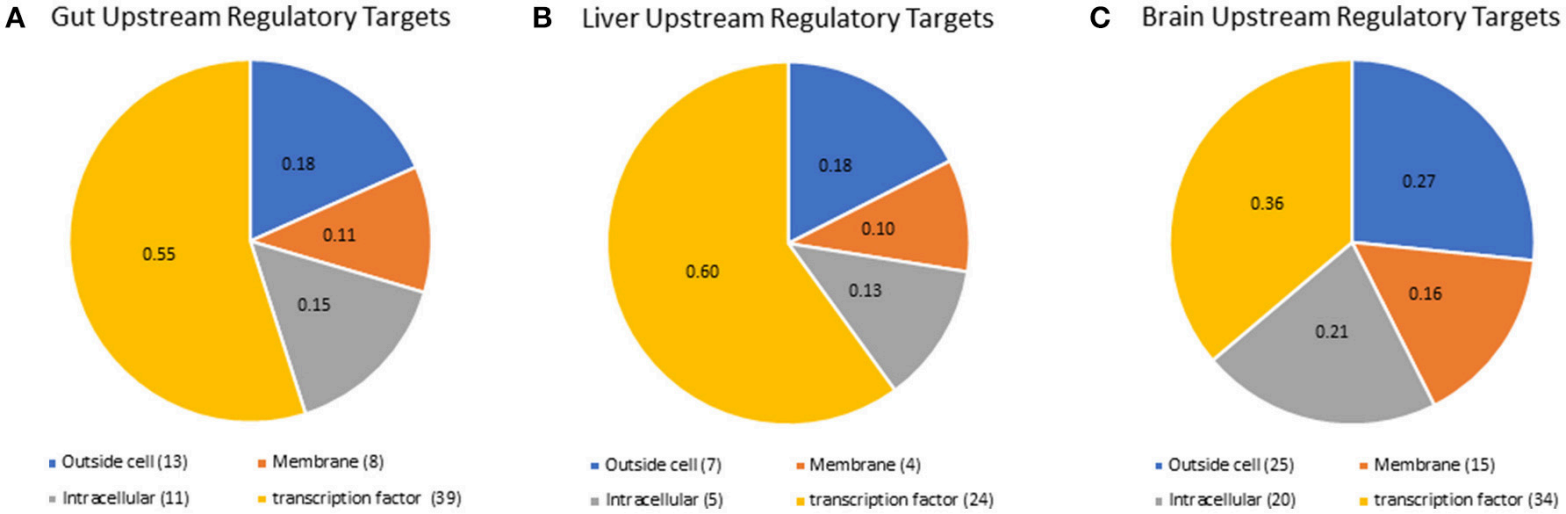
Pathway studio representation of expression targets identified for **(A)** gut, **(B)**, liver, and **(C)** brain. All of the expression targets identified in any tissue were mapped by pathway studio to their cellular location by the GO categories. The ID's for the expression targets are found in Supplementary Table 6.

Although we did not detect mRNA or proteins for all nuclear receptors, we were able to predict which nuclear receptors and transcription factors would be expected to regulate downstream gene expression in each tissue, using the RNA-seq results in a more holistic, network-based approach. Lack of detection of nuclear receptors is a common result due to their poor stoichiometry and this supports the use of network-based analyses to delineate nuclear receptor-mediated signaling mechanisms. We also identified upstream regulatory targets, including transcritption factors and signaling pathway components, that were likely to drive the expression of the genes that were highly expressed in each tissue (Fold Change > 2). A list containing all of the gene symbols and names for transcriptional regulators identified is available in supplemental information (Supplementary Table 6). For the gut tissue, 79 expression targets were identified (Figure 4A), 49 expression targets were identified in the liver tissue (Figure 4B), and there were 106 combined expression targets for the hypothalamus and telencephalon (Figure 4C). The liver and gut shared more expression targets (17) than either the liver and brain (2) or the gut and brain (2). Only two expression targets were shared among all tissues, which were two isoforms of fibroblast growth factor (FGF), a mediator of differentiation and development of numerous cell types throughout the body (51). Interestingly, in the gut and liver, the majority of the upstream regulatory targets were nuclear transcription factors (48% gut, 47% liver, 31% brain); however, in the brain a higher proportion of the upstream regulatory targets were extracellular proteins and ligands (18% for gut and liver and 27% for brain), or membrane receptors (26% gut, 22% liver, 38% brain). These data are intriguing given the growing appreciation for the importance of membrane receptors and endocrine ligands and their signaling mechanisms in the brain, particularly for neuroendocrine functions and responses to endocrine modulators such as ethinylestradiol or levonorgestrel (28, 52).

### Confirmation of RNAseq transcript data with quantitative PCR

**Figure 5:**
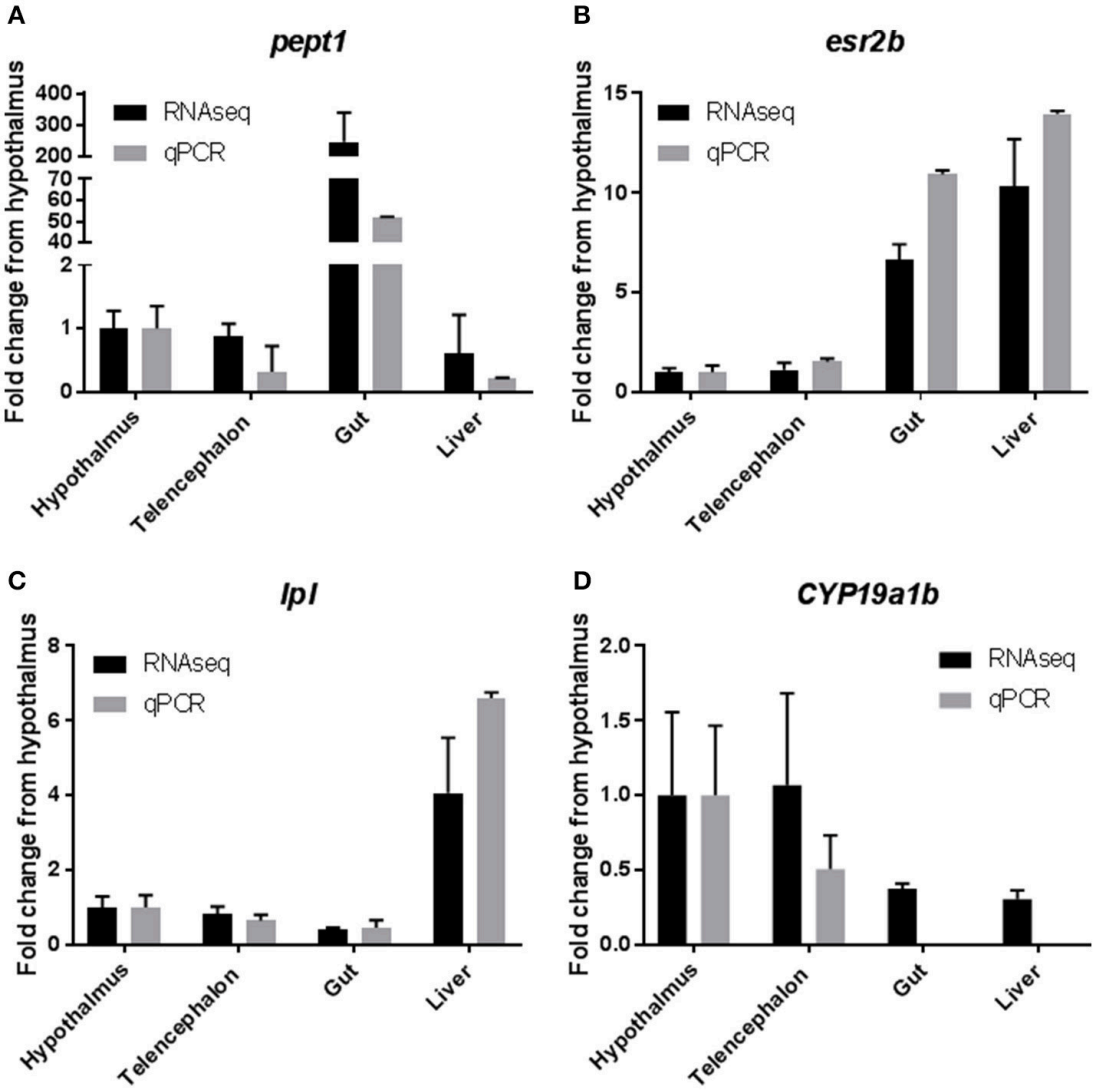
Confirmation of RNAseq results with quantitative PCR. Selected genes from the RNAseq data set were used to confirm the results by qPCR. Results are presented as mean ± standard deviation fold change from the hypothalamus tissue. **(A)** Peptide Transporter 1 (*pept1*). **(B)** Estrogen Receptor 2b (*esr2b*). **(C)** Lipoprotein Lipase (*lpl*). **(D)** Cytochrome P450 19a1b (*cyp19a1b*).

Results from qPCR analysis of select tissue specific transcrips indicated good agreement between RNAseq data and qPCR. RNAseq data indicated that Peptide transporter 1 (*pept1*), a transporter that is responsible for moving small polypeptides from the gastrointestinal lumen into the gastrointestinal system, was highly expressed (>200 fold) in the gastrointestinal tissues, when compared to all other tissues (36). Results from the qPCR analysis confirmed this finding with a >50 fold increase in expression in the gastrointestinal tissue (Figure 5A). Expression of estrogen receptor 2b (*esr2b*) was found to be highest in the liver tissues, followed closely by the gut tissue, with much lower expression in the brain tissues, which was mirrored by the qPCR data (Figure 5B) and as mentioned above by the work of Filby and Tyler (50). Expression of lipoprotein lipase (*lpl*), an enzyme responsible for lipid digestion, was increased 4 fold in the liver when compared to other tissues, which was found to be similar in the qPCR data as well (Figure 5C). Finally, expression of *cyp19a1b* (aromatase b), an enzyme responsible for conversion of testosterone to estradiol, was high in the brain tissues from the RNAseq datasets, with very little expression in the liver and gut (53). These data were also confirmed by qPCR with high levels of expression in the hypothalamus and telencephalon and no detectable levels of expression of this transcript in the liver or gut (Figure 5D).

### Tissue-specific protein expression

Protein identification and differential expression were computed using proteomics specific algorithms, such as Protein Pilot. We obtained 150,150 spectra from the 10 protein salt fractions eluted from the SCX column, and we were able to identify 26,396 distinct peptides at a 1% global FDR, which resulted in the identification of 4,045 protein groups at a 1% global FDR. Of note, a tradeoff exists between database comprehensiveness and redundancy. Only 40% of identified protein groups had unambiguous identifications, suggesting a high level of redundancy in the database. This high level of redundancy is expected because the database consists of both FHM and zebrafish sequences (Supplementary Figure 2A), and the peptide dynamic range was calculated by ProteinPilot to span 2.95 orders of magnitude.

Overall, an average of 3,840 (range of 3,838–3,841) proteins were quantified in each tissue (Supplementary Figure 2B). Of those, 69.76% (69.05–69.92%) were supported with enough evidence to calculate a *p*-value testing the hypothesis that differences observed in iTRAQ label ratios were random. The median log ratio for gut tissue was consistent across both replicates; however, there was a bit of variability between the telencephalon (−0.02 and 0.16) and liver (0.09 and 0.33) replicates. Consistency amongst replicates was the highest for the liver and gut, and lowest for the telencephalon (Supplementary Figure 2C).

**Figure 6:**
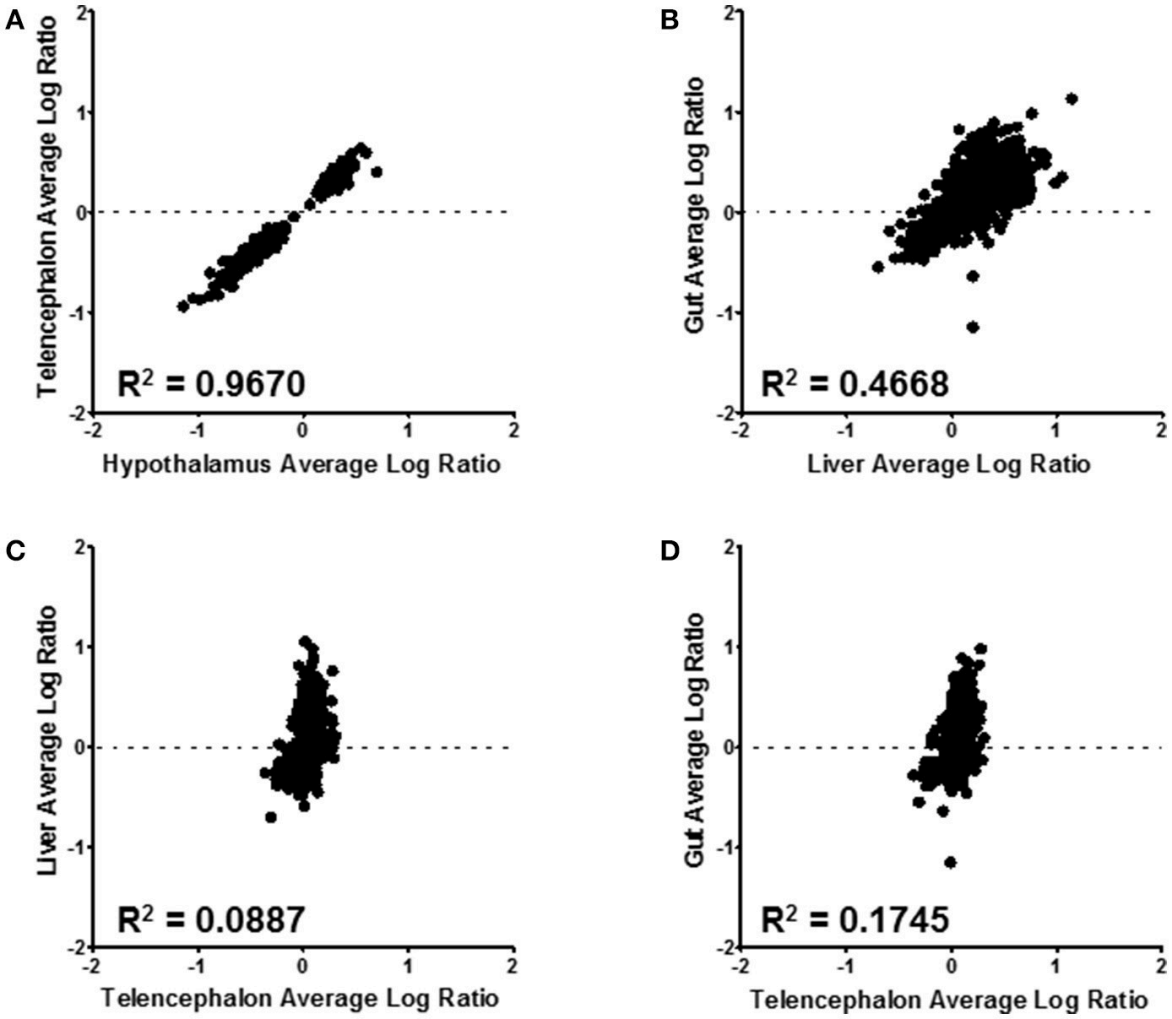
Tissue level comparisons of all confidently quantified protein ratios. All tissue comparisons had a *p*-value ≤ 0.001. Hypothalmus to telencephalon correlation was made by using the liver as the denominator of the ratio calculation **(A)**; while the gut to liver **(B)**, liver to telencephalon **(C)**, and gut to telencephalon **(D)** were made using the hypothalamus as the normalizing tissue. The *R*^2^ value for each correlation is displayed on the corresponding graph.

Correlations between expressed proteins among the tissues is shown in Figure 6. The most similar were the hypothalamus and telencephalon, with an *R*^2^ value of 0.967 (Figure 6A). This was expected as there are small differences in structural proteins among different parts of the brain. Comparing proteins of the gut with the liver shows an *R*^2^ value of 0.467 (Figure 6B). These were the second most similar comparison. There was little similarity between telencephalon and liver (*R*^2^ = 0.089) (Figure 6C) or between telencephalon and gut (*R*^2^ = 0.175) (Figure 6D), underscoring the different functions of these disparate tissues.

As previously mentioned, the hypothalamus and telencephalon had a high degree of similarity; however, there were some important differences noted. Specifically, glial fibrillary acidic protein (GFAP) was higher in the telencephalon than in the hypothalamus, while neurofilament medium polypeptide (NEFM) was higher in the hypothalamus. GFAP is an intermediate filament protein that is synthesized only in astrogliocytes in the brain. It provides cytoskeletal structure for these cells and has a critical role in their activation when the brain becomes injured through disease or from traumatic brain injury (54). Our data suggests that there may be more astroglial cells in the telencephalon than in the hypothalamus. NEFM is a member of the neurofilament family consisting of light, medium and heavy neurofilaments. These are the major structural components of axons (55) and are responsible for the radial growth of the axon. It is clear now that NEFM respond to a myelin signal, probably through a phosphorylation cascade (55). Our results suggest that in fathead minnows, the hypothalamus contains more long axons than the telencephalon. This may facilitate longer-range interactions between neurons.

SNEA analysis was clearly able to differentiate tissue-specific biological functions enriched with the proteins identified in the iTRAQ experiment. In the gut, 37 subnetworks were found to be enriched including intestinal barrier, intestine function and lipid adsorption (Supplementary Table 7). In the liver, 37 subnetworks were identified including detoxification, xenobiotic clearance, liver metabolism, and liver function (Supplementary Table 8). The genes that were higher in the telencephalon and hypothalamus were combined into a single list for the brain, which was used for SNEA. The analysis identified over 100 subnetworks including neurotransmitter secretion, synaptic transmission, regeneration, and brain function (Supplementary Table 9).

### Comparison of RNA-seq with proteomics

**Figure 7:**
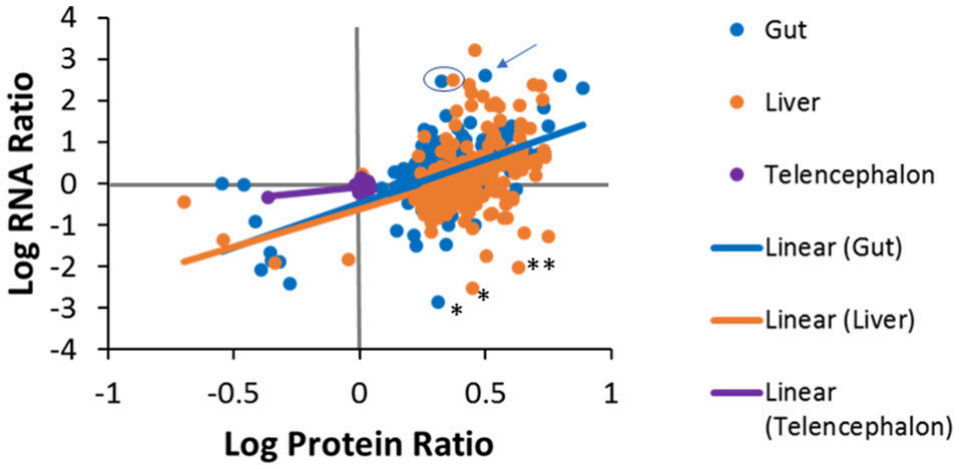
Tissue specific correlations between protein log ratios and RNA log ratios for genes that were identified in both experiments. Solid lines are tissue-specific regression lines. Examples discussed in the manuscript text are denoted with the following symbols; ^*^ fatty acid binding protein 7 (fabp7), ^**^ (gapdh), the arrow points to dipeptidase 1 (dpep1) and the blue circle encloses carboxypeptidase A1 (cpa1) in the liver and the gut.

Pairwise comparisons were made to investigate the level of agreement between transcript log ratios obtained from RNA-seq and protein log ratios obtained from iTRAQ. The pairwise comparisons made at human homolog level are shown in Figure 7. We had expected to see a positive correlation for each entity between RNA-seq and proteomics for each tissue, but, as can be observed, this is not the case for all genes. A positive log ratio for RNA expression, with a negative log ratio for proteins was not observed in any tissue. In the telencephalon, most log ratios are close to zero as there were few differences from the hypothalamus detected by either RNA-seq or iTRAQ. In the liver, about half (59%) of the genes were in agreement, while the other half had positive protein log ratios and negative RNA log ratios. In the gut, 69% of the genes were in agreement and only 31% had positive protein log ratios and negative RNA log ratios. The slopes of the regression lines are 0.662 (R^2^ = 0.2912), 1.831 (R^2^ = 0.141), and 2.133 (R^2^ = 0.324) for the telencephalon, liver, and gut, respectively. Some of the variation could be due to ratio compression, a well-known artifact of iTRAQ proteomics (56, 57) given that these slopes are similar to those observed in these other studies.

Additionally, differences between protein and RNA levels for specific genes could be due to differential regulation in translation or turnover rates of protein and/or its transcript. For example, in the liver and the gut, fatty acid binding protein 7 (fabp7) had positive protein log ratios but negative RNA log ratios. These data suggest that the liver and gut have more fabp7 protein than the hypothalamus while there is more message in the hypothalamus (Figure 7). The common qPCR reference gene, glyceraldehyde 3-phosphate dehydrogenase (gapdh) also had higher protein levels in the liver compared to the hypothalamus, but less message. Alternatively, there were many cases in which the protein ratios in the liver or gut were positive, but much less than the ratio for RNA. Some examples are fatty acid binding protein 2 (fabp2), dipeptidase 1 (dpep1), and annexin 2 (anxa2) in the gut, carboxypeptidase A1 (cpa1) in the liver and gut, and the fibrinogen subunits (fga, fgg, fgb), 3-oxoacid CoA-transferase 1 (oxct1), urate oxidase (uox), and tetratricopeptide repeat domain 36 (ttc36) in the liver. Conversely, some genes exhibited high protein expression, but low RNA expression. A similar phenomenon has been seen in plants where iron deficiency results in increased protein expression of members of the conserved eukaryotic elongation factor 5A (eIF5A) family without a concordant increase in mRNA abundance (58). This can also be explained by differential half-lives, i.e. the half-life of a protein can be much longer than that of the RNA, as is the case for ribosomal proteins. There are roughly ten million ribosomes per eukaryotic cell and they are fairly stable compared to the half-lives of mRNAs for the ribosomal proteins, which are fairly short by comparison (59). Proteomics and transcriptomics measurements are made on increases or decreases from the steady state level of these molecules in tissues, which is quite different for mRNA and protein for ribosomes. Further investigations will be needed to determine if variations are an artifact of iTRAQ ratio compression or a true difference in the magnitude of expression.

**Figure 8:**
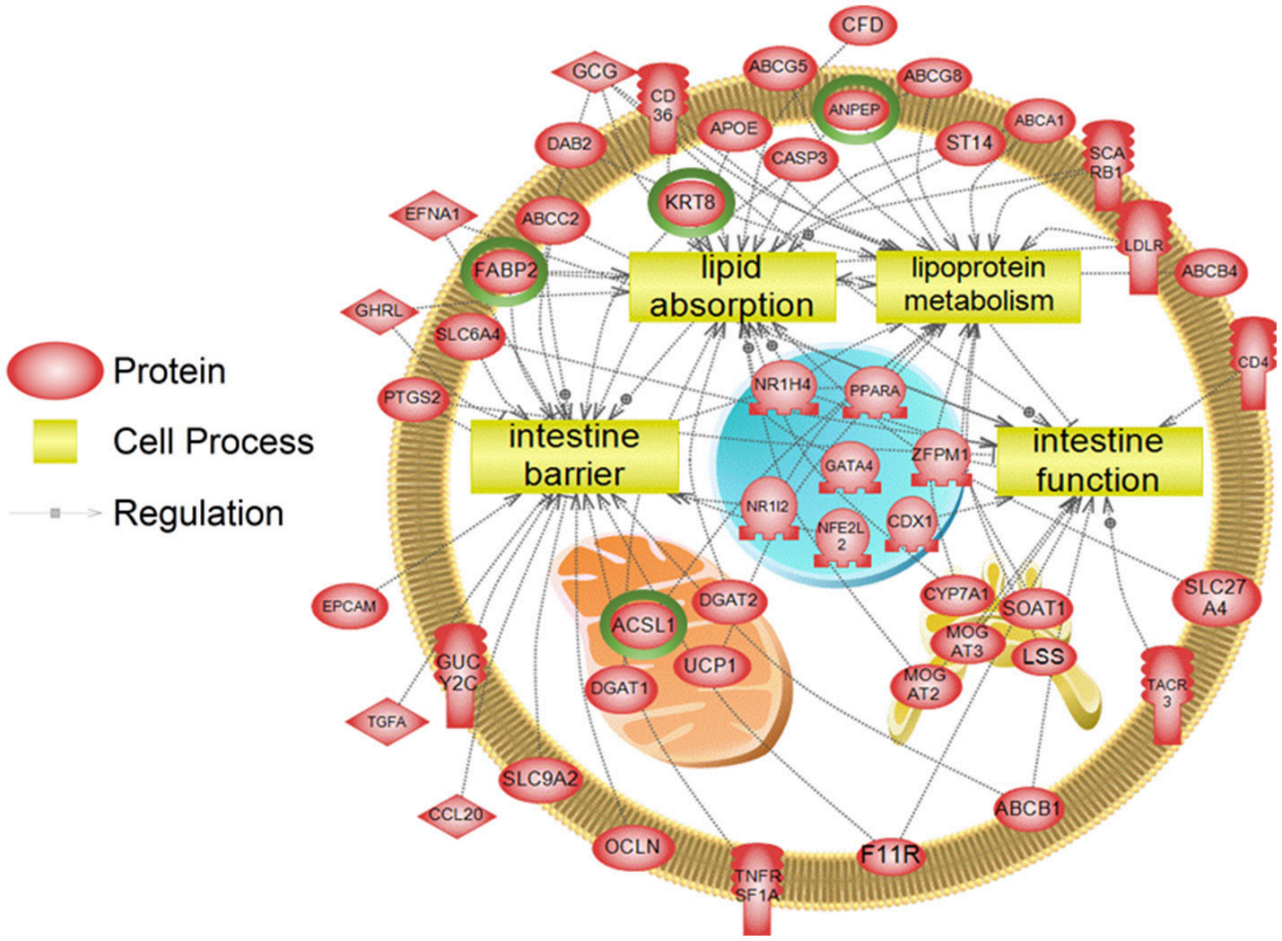
Subnetwork enrichment analysis of the transcriptome for the gut. The figure represents the joining of 4 top pathways identified by the analysis. Genes encircled in green were also found to be enriched in the gut in the proteomics experiment. ABCA1, ATP-binding cassette, sub-family A (ABC1), member 1; ABCB1, ATP-binding cassette, sub-family B (MDR/TAP), member 1; ABCB4, ATP-binding cassette, sub-family B (MDR/TAP), member 4; ABCC2, ATP-binding cassette, sub-family C (CFTR/MRP), member 2; ABCG5, ATP-binding cassette, sub-family G (WHITE), member 5; ABCG8, ATP-binding cassette, sub-family G (WHITE), member 8; ACSL1, acyl-CoA synthetase long-chain family member 1; ANPEP, alanyl (membrane) aminopeptidase; APOE, apolipoprotein E; CASP3, caspase 3, apoptosis-related cysteine peptidase; CCL20, chemokine (C-C motif) ligand 20; CD36, CD36 molecule (thrombospondin receptor); CD4, CD4 molecule; CDX1, caudal type homeobox 1; CFD, complement factor D (adipsin); CYP7A1, cytochrome P450, family 7, subfamily A, polypeptide 1; DAB2, disabled homolog 2, mitogen-responsive phosphoprotein; DGAT1, diacylglycerol O-acyltransferase 1; DGAT2, diacylglycerol O-acyltransferase 2; EFNA1, ephrin-A1; EPCAM, epithelial cell adhesion molecule; F11R, F11 receptor; FABP2, fatty acid binding protein 2, intestinal; GATA4, GATA binding protein 4; GCG, glucagon; GHRL, ghrelin/obestatin prepropeptide; GUCY2C, guanylate cyclase 2C (heat stable enterotoxin receptor); KRT8, keratin 8; LDLR, low density lipoprotein receptor; LSS, lanosterol synthase (2,3-oxidosqualene-lanosterol cyclase); MOGAT2, monoacylglycerol O-acyltransferase 2; MOGAT3, monoacylglycerol O-acyltransferase 3; NFE2L2, nuclear factor (erythroid-derived 2)-like 2; NR1H4, nuclear receptor subfamily 1, group H, member 4; NR1I2, nuclear receptor subfamily 1, group I, member 2; OCLN, occludin, PPARA, peroxisome proliferator-activated receptor alpha; PTGS2, prostaglandin-endoperoxide synthase 2 (prostaglandin G/H synthase and cyclooxygenase), SCARB1, scavenger receptor class B, member 1; SLC27A4, solute carrier family 27 (fatty acid transporter), member 4; SLC6A4, solute carrier family 6 (neurotransmitter transporter, serotonin), member 4; SLC9A2, solute carrier family 9, subfamily A (NHE2, cation proton antiporter 2), member 2; SOAT1, sterol O-acyltransferase 1; ST14, suppression of tumorigenicity 14 (colon carcinoma); TACR3, tachykinin receptor 3, TGFA, transforming growth factor, alpha; TNFRSF1A, tumor necrosis factor receptor superfamily, member 1A, UCP1, uncoupling protein 1 (mitochondrial, proton carrier), ZFPM1, zinc finger protein, multitype 1.

**Figure 9:**
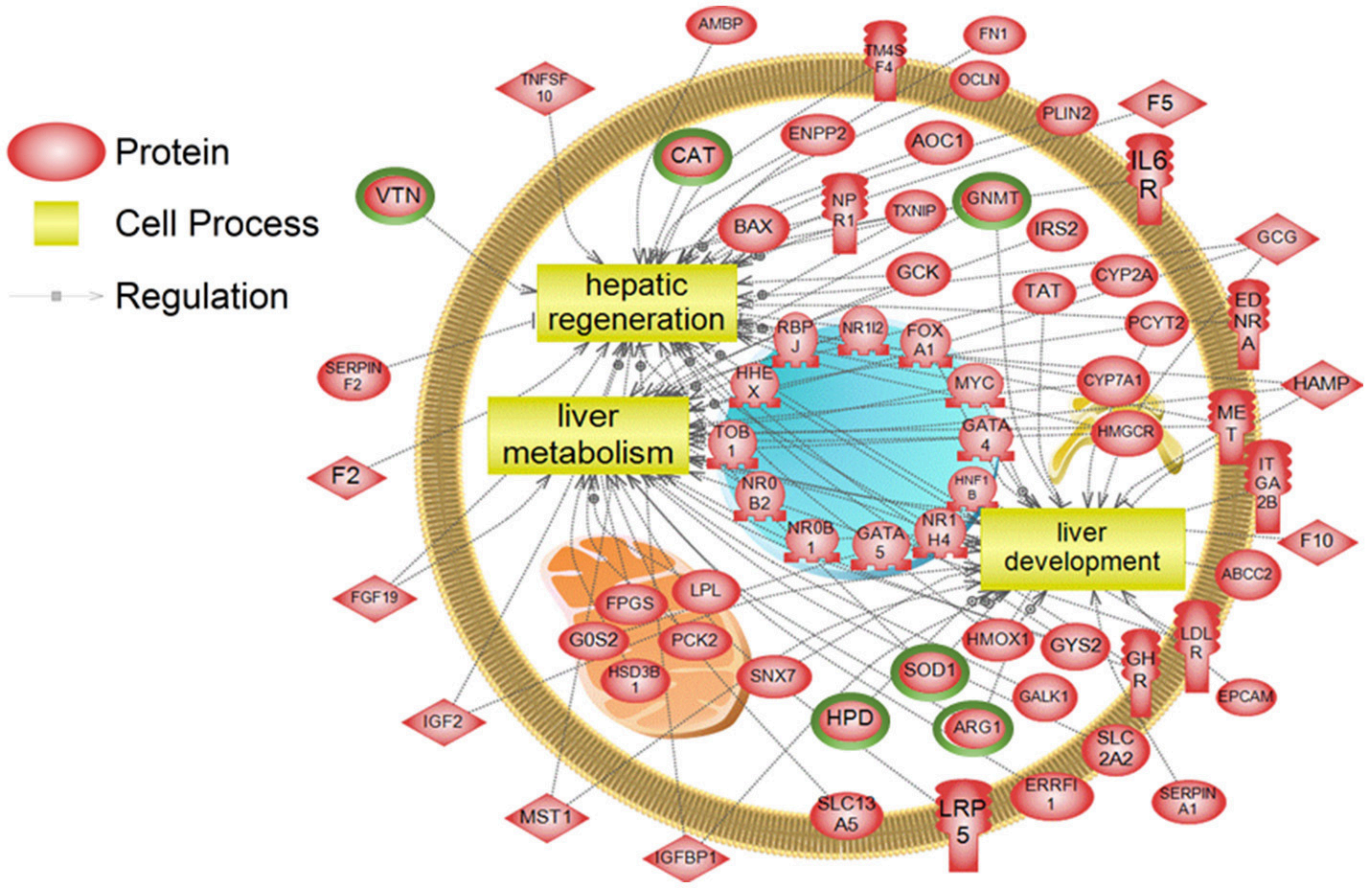
Subnetwork enrichment analysis of the transcriptome for the liver. The figure represents the joining of 3 top pathways identified by the analysis. Genes encircled in green were also found to be enriched in the liver in the proteomics experiment. ABCC2, ATP-binding cassette, sub-family C (CFTR/MRP), member 2; AMBP, alpha-1-microglobulin/bikunin precursor; AOC1, amiloride binding protein 1 [amine oxidase (copper-containing)]; ARG1,arginase, liver; BAX, BCL2-associated X protein; CAT, catalase; CYP2A, cytochrome P450, family 2, subfamily A; CYP7A1, cytochrome P450, family 7, subfamily A, polypeptide 1; EDNRA, endothelin receptor type A; ENPP2, ectonucleotide pyrophosphatase/phosphodiesterase 2; EPCAM, epithelial cell adhesion molecule; ERRFI1, ERBB receptor feedback inhibitor 1; F10, coagulation factor X; F2, coagulation factor II (thrombin); F5, coagulation factor V (proaccelerin, labile factor); FGF19, fibroblast growth factor 19; FN1, fibronectin 1; FOXA1, forkhead box A1; FPGS, olylpolyglutamate synthase; G0S2, G0/G1switch 2; GALK1, galactokinase 1; GATA4, GATA binding protein 4; GATA5, GATA binding protein 5; GCG, glucagon; GCK, glucokinase (hexokinase 4); GHR, growth hormone receptor; GNMT, glycine N-methyltransferase; GYS2, glycogen synthase 2 (liver); HAMP, hepcidin antimicrobial peptide; HHEX, hematopoietically expressed homeobox; HMGCR, 3-hydroxy-3-methylglutaryl-CoA reductase; HMOX1, heme oxygenase (decycling) 1; HNF1B, HNF1 homeobox B; HPD, 4-hydroxyphenylpyruvate dioxygenase; HSD3B1, hydroxy-delta-5-steroid dehydrogenase, 3 beta- and steroid delta-isomerase 1; IGF2, insulin-like growth factor 2 (somatomedin A); IGFBP1, insulin-like growth factor binding protein 1; IL6R, interleukin 6 receptor; IRS2, insulin receptor substrate 2; ITGA2B, integrin, alpha 2b (platelet glycoprotein IIb of IIb/IIIa complex, antigen CD41); LDLR, low density lipoprotein receptor; LPL, lipoprotein lipase; LRP5, low density lipoprotein receptor-related protein 5; MET, met proto-oncogene (hepatocyte growth factor receptor); MST1, macrophage stimulating 1 (hepatocyte growth factor-like); MYC, v-myc myelocytomatosis viral oncogene homolog (avian); NPR1, natriuretic peptide receptor A/guanylate cyclase A (atrionatriuretic peptide receptor A); NR0B1, nuclear receptor subfamily 0, group B, member 1; NR0B2, nuclear receptor subfamily 0, group B, member 2; NR1H4, nuclear receptor subfamily 1, group H, member 4; NR1I2, nuclear receptor subfamily 1, group I, member 2; OCLN, occludin; PCK2, phosphoenolpyruvate carboxykinase 2 (mitochondrial); PCYT2, phosphate cytidylyltransferase 2, ethanolamine; PLIN2, perilipin 2; RBPJ, recombination signal binding protein for immunoglobulin kappa J region; SERPINA1, serpin peptidase inhibitor, clade A (alpha-1 antiproteinase, antitrypsin), member 1; SERPINF2, serpin peptidase inhibitor, clade F (alpha-2 antiplasmin, pigment epithelium derived factor), member 2; SLC13A5, solute carrier family 13 (sodium-dependent citrate transporter), member 5; SLC2A2, solute carrier family 2 (facilitated glucose transporter), member 2; SNX7, sorting nexin 7; SOD1, superoxide dismutase 1, soluble; TAT, tyrosine aminotransferase; TM4SF4, transmembrane 4 L six family member 4; TNFSF10, tumor necrosis factor (ligand) superfamily, member 10; TOB1, transducer of ERBB2, 1; TXNIP, thioredoxin interacting protein; VTN, vitronectin.

**Figure 10:**
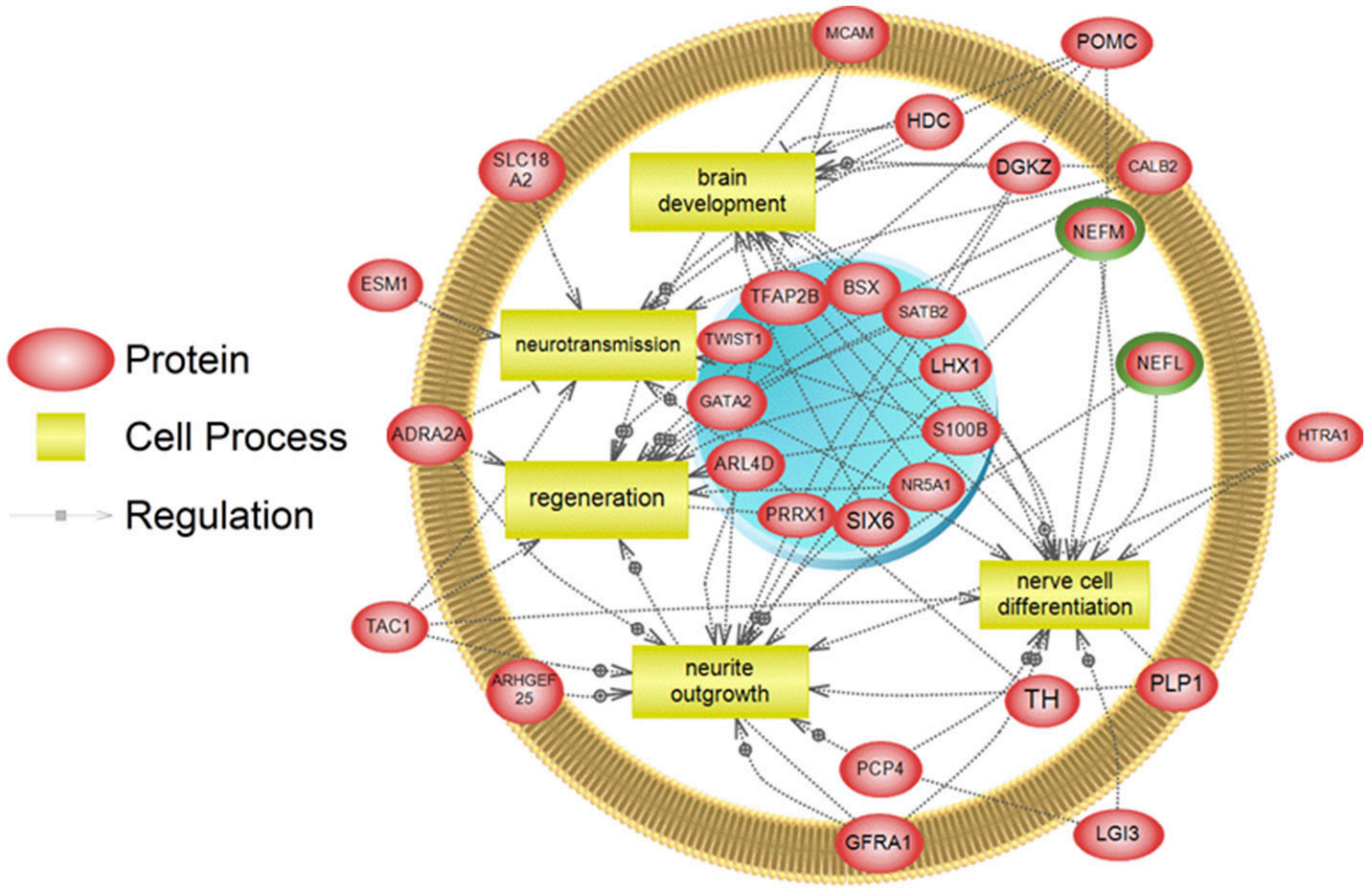
Subnetwork enrichment analysis of the transcriptome for the brain. The figure represents the joining of 5 top pathways identified by the analysis. Genes encircled in green were also found to be enriched in the brain in the proteomics experiment. ADRA2A, adrenoceptor alpha 2A; ARHGEF25, Rho guanine nucleotide exchange factor (GEF) 25; ARL4D, ADP-ribosylation factor-like 4D; BSX, brain-specific homeobox; CALB2, calbindin 2; DGKZ, diacylglycerol kinase, zeta 104kDa; ESM1, endothelial cell-specific molecule 1; GATA2, GATA binding protein 2; GFRA1, GDNF family receptor alpha 1; HDC, histidine decarboxylase; HTRA1, HtrA serine peptidase 1; LGI3, leucine-rich repeat LGI family, member 3; LHX1, LIM homeobox 1; MCAM, melanoma cell adhesion molecule; NEFL, neurofilament, light polypeptide; NEFM, neurofilament, medium polypeptide; NR5A1, nuclear receptor subfamily 5, group A, member 1; PCP4, Purkinje cell protein 4; PLP1, proteolipid protein 1; POMC, proopiomelanocortin; PRRX1, paired related homeobox 1; S100B, S100 calcium binding protein B; SATB2, SATB homeobox 2; SIX6, SIX homeobox 6; SLC18A2, solute carrier family 18 (vesicular monoamine), member 2; TAC1, tachykinin, precursor 1; TFAP2B, transcription factor AP-2 beta (activating enhancer binding protein 2 beta); TH, tyrosine hydroxylase; TWIST1,Twist homolog 1.

To examine higher order similarities and differences between the tissue RNA-seq and proteomics datasets, we utilized PathwayStudio^TM^'s SNEA on genes and proteins, which measured at least 2-fold higher than in the hypothalamus tissue. A comprehensive list of subnetworks enriched in the RNA-seq and proteomic datasets in each tissue is provided in Supplementary Tables 2–5, 7–9. Of note, there was overlap in enriched cell processes between transcriptomic and proteomic datasets from each respective tissue. Specifically, there were 8 cell processes common across both datasets in the gut. A subset of these shared cell processes is shown in Figure 8 all of which are processes that would be expected in the gut, including lipid absorption, lipoprotein metabolism, intestinal barrier function, and general intestinal function. For the liver datasets, 3 common cell processes were found to be enriched and all were related to liver function including hepatic regeneration, liver metabolism, and liver development (Figure 9). Finally, when comparing enriched cell processes in the brain between the RNA and protein datasets, 21 cell processes are common between the two datasets. A subset of these process is given in Figure 10, which demonstrates enrichment of brain development, neurotransmission, regeneration, neurite outgrowth, and nerve cell differentiation. If we examine genes/proteins associated with these overlapping enriched cell processes, we find that only a few are conserved among the two datasets for each tissue, which are circled in green (Gut: 3, Liver: 5, Brain: 2).

Taken as a whole, the RNA and protein datasets identified numerous cell processes that are unique to each dataset. Overlapping cell processes were typically those specific to each tissue, indicating that both measurements are likely to converge on cell processes and functions that are strongly associated with those specific tissues despite very few individual genes/proteins coinciding between the two datasets.

### Relationship of findings to endocrinology

It is important for researchers to understand the tissue-specific expression of receptors for peptide and steroid-based hormones. The database we have created by combining the PacBio data set with multiple Illumina RNA-seq data sets will enable researchers to find sequences for genes of interest that may propel their research to a new level. As mentioned above, our data for esr2a and esr2b matched perfectly to data obtained by Northern blots (50), thus indicating that the RNA-seq data, despite going through an amplification scheme, closely matches the actual relative concentrations of important genes.

## Conclusions

This study is the first to apply single DNA molecule sequencing to generate a transcriptome for FHM. This transcriptome was made up of transcripts from whole brain, gut, liver, gonad, heart, gill, head kidney, and trunk kidney and is robust. It will serve as a good scaffold for future transcriptomics and proteomics projects and may have some utility to help with the FHM genome annotation. In addition, we mapped tissue-specific genes for gut, liver, hypothalamus and telencephalon proteomes and transcriptomes in order to identify and characterize their specific components in each tissue to highlight the utility of our transcriptomic and proteomic sequence databases and to identify cellular pathways enriched during homeostasis that may inform relevant endpoints in future ecotoxicogenomic studies in the ecologically relevant FHM. Our results showed that both the transcriptomes and the proteomes differed by tissue, with the hypothalamus and the telencephalon presenting the highest degree of similarity. The transcriptomic and proteomic sequence information generated in this study will be invaluable in future functional genomic studies investigating the effects of endocrine disrupting chemicals present in the environment on endocrine active tissues of the ecologically-relevent FHM, particularly the neuro-endocrine ssytem. The data is publicly available.

## Data availability

RNAseq data can be found at GEO with accession # GSE119871.

Proteomics data sets have been submitted to the ProteomeXchange Consortium via PRIDE with the dataset identifier PXD010216.

Proteomics information for the identification of proteins/peptides from mass spectra will be supplied as an excel spreadsheet upon request by ND.

## Ethics statement

This study was carried out in accordance with the recommendations of the University of Florida IACUC committee. The protocol was approved by the University of Florida IACUC committee.

## Author contributions

JB, NG-R, TS-A, and ND conceived of the project, helped with analysis and writing of the manuscript. CL, LS, and JB performed the experiments, analyzed data, and contributed to the writing of the manuscritpt. FY performed bioinformatics analysis and annotation for long reads from the PacBio instrument. He also performed the RNA-seq analysis. CS-S performed the iTRAQ experiments by LC MS/MS. CL performed bioinformatics and statistical analysis of the proteomics data and the RNA-seq data. AB and JB performed the qPCR analysis. DM-A discussed experimental strategy and performed the PACBio sequencing. CL, LS, JB, DM-A, FY, CS-S, AB, TS-A and ND wrote sections of the manuscript and all authors have read and approved the submitted version.

### Conflict of interest statement

The authors declare that the research was conducted in the absence of any commercial or financial relationships that could be construed as a potential conflict of interest.

## Supplementary material

Supplementary Figure 1 Pairwise comparisons for differential transcript expression were made for each tissue; hypothalamus to telencephalon **(A)**, hypothalamus to liver **(B)**, liver to telencephalon **(C)**, gut to telencephalon **(D)**, gut to hypothalamus **(E)**, and gut to liver **(F)**. Black dots represent transcripts that were compared and red dots represent transcripts that were found to be statistically different at the 5% FDR cutoff. The data points forming a column on the left most portion of the graph represent transcripts that were measured in only one of the tissues being compared.

Supplementary Figure 2 Quality metrics for iTRAQ data and protein identification. **(A)** Ambiguity was assessed at both the level of protein. **(B)** The number of proteins that we quantified, quantified confidently, and the median log ratio for each iTRAQ label were assessed. **(C)** Correlations coefficients (r) between individual iTRAQ labeled samples are displayed.

Supplementary Table 1 Primer sequences, sources, and efficiencies for qPCR analysis.

Supplementary Table 2 Transcriptomics SNEA illustrating regulation of cell processes in the gut.

Supplementary Table 3 Transcriptomics SNEA illustrating regulation of cell processes in the liver.

Supplementary Table 4 Transcriptomics SNEA illustrating regulation of cell processes in the telencephalon.

Supplementary Table 5 Transcriptomics SNEA illustrating regulation of cell processes in the hypothalamus.

Supplementary Table 6 Expression targets derived from Pathway Studio for gut, liver and brain. These are the genes highlighted in Figure 4.

Supplementary Table 7 Proteomics SNEA results for regulation of cell processes in the gut.

Supplementary Table 8 Proteomics SNEA results for regulation of cell processes in the liver.

Supplementary Table 9 Proteomics SNEA results for regulation of cell processes in the brain.
