# Supplementary figures and images for: Tissue-Based Mapping of the Fathead Minnow (Pimephales promelas) Transcriptome and Proteome
Source: Front Endocrinol (Lausanne). 2018 Nov 6;9:611. doi: 10.3389/fendo.2018.00611 (PMC6232228; doi:10.3389/fendo.2018.00611)

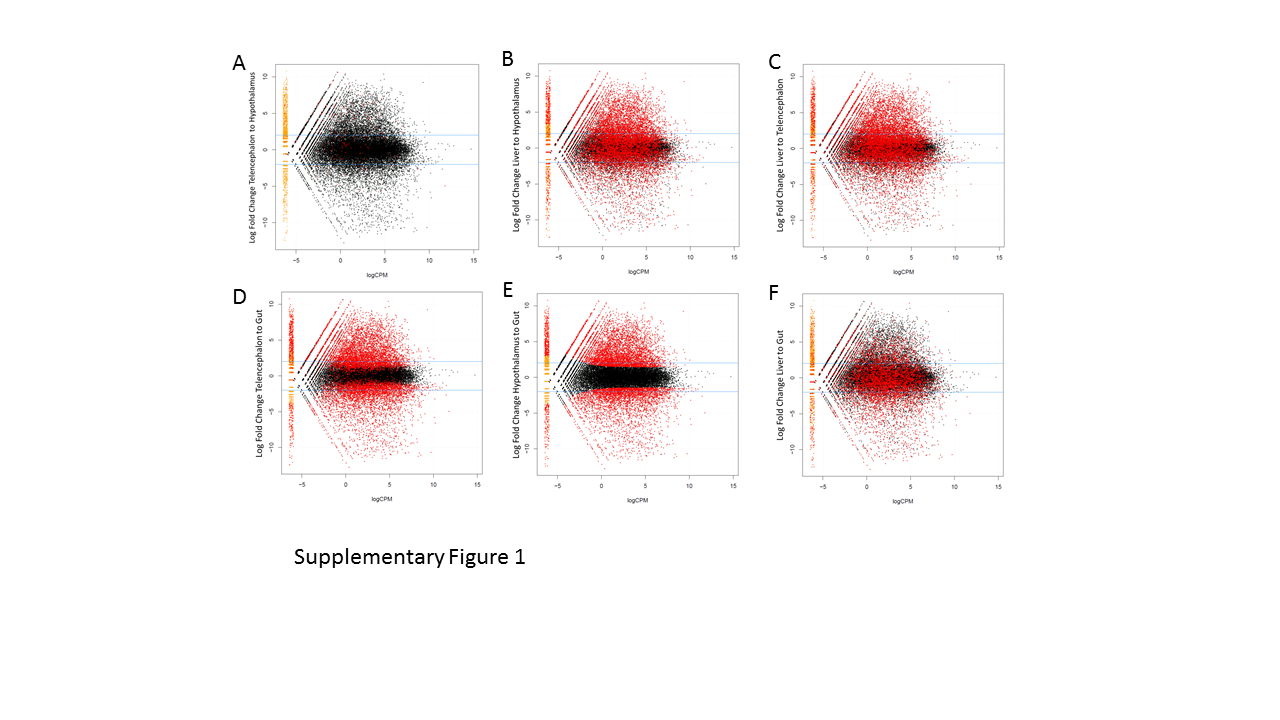

Supplement: Supplementary Figure 1 — Pairwise comparisons for differential transcript expression were made for each tissue; hypothalamus to telencephalon (A), hypothalamus to liver (B), liver to telencephalon (C), gut to telencephalon (D), gut to hypothalamus (E), and gut to liver (F). Black dots represent transcripts that were compared and red dots represent transcripts that were found to be statistically different at the 5% FDR cutoff. The data points forming a column on the left most portion of the graph represent transcripts that were measured in only one of the tissues being compared. [file Image_1.TIF]

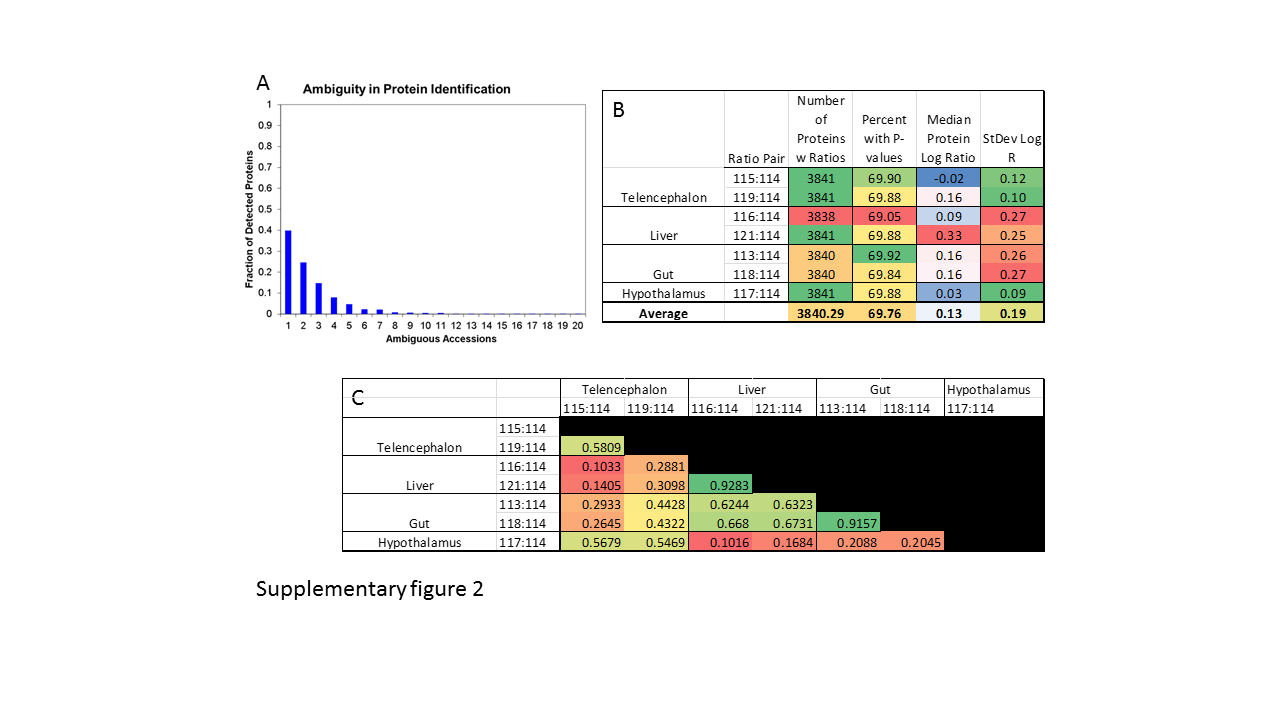

Supplement: Supplementary Figure 2 — Quality metrics for iTRAQ data and protein identification. (A) Ambiguity was assessed at both the level of protein. (B) The number of proteins that we quantified, quantified confidently, and the median log ratio for each iTRAQ label were assessed. (C) Correlations coefficients (r) between individual iTRAQ labeled samples are displayed. [file Image_2.TIF]
