# Supplementary material for: Tissue-Based Mapping of the Fathead Minnow (Pimephales promelas) Transcriptome and Proteome
Source: Front Endocrinol (Lausanne). 2018 Nov 6;9:611. doi: 10.3389/fendo.2018.00611 (PMC6232228; doi:10.3389/fendo.2018.00611)
Supplement: Supplementary Table 1 — Primer sequences, sources, and efficiencies for qPCR analysis. [file Presentation_1.pdf]

Supplementary Information

Tissue-based mapping of the fathead minnow (*Pimephales promelas*) transcriptome and proteome

Candice Lavelle[a,c*], L. Cody Smith[b,c*], Joseph H Bisesi[a,c,*], Fahong Yu[d], Cecilia Silva-Sanchez[cd], David Moraga-Amador[d], Amanda N. Buerger[a,c], Natàlia Garcia-Reyero[e], Tara Sabo-Attwood[a,c], Nancy D. Denslow[b,c]

a. Department of Environmental and Global Health, University of Florida, Gainesville, FL, USA
b. Department of Physiological Sciences, University of Florida, Gainesville, FL
c. Center for Environmental and Human Toxicology, University of Florida, Gainesville, FL, USA
d. Interdisciplinary Center for Biotechnology Research, University of Florida, Gainesville, FL, USA
e. Environmental Laboratory, US Army Engineer Research & Development Center, Vicksburg, MS, USA

*Co-first authors

Supplementary Table 1: Primer sequences, sources, and efficiencies for qPCR analysis

Gene	Efficiency	R^2	Temperature (°C)	F Sequence (5'-3')	R Sequence (5'-3')	Source
cyp19a1b	101.90%	0.98	58	CGCTCAGTGGGATTTTTAGG	GGCATCATCTTCAACAGCAA	Martyniuk et al. 2010
esr2b	93.30%	0.998	60	GCCACCTCCAGATTCAG	CACGACTCTCCACACCTTCAG	Filby et al. 2006
lpl	108.40%	0.998	58	CTGTGACCTCCAGAACACTATG	GAGTCGATGAACAGGTGGATG	Bisesi et al. 2015
pept1	102.40%	0.997	58	ACACCGCAGTAAGCAATACC	ACCTTCAGTGCCATCTTTACC	Bisesi et al. 2015

Filby, A. L., Thorpe, K. L., & Tyler, C. R. (2006). Multiple molecular effect pathways of an environmental oestrogen in fish. *Journal of Molecular Endocrinology*, *37*(1), 121-134.

Bisesi, J., Ngo, T., Ponnavolu, S., Liu, K., Lavelle, C., Afrooz, A. R. M., ... & Sabo-Attwood, T. (2015). Examination of single-walled carbon nanotubes uptake and toxicity from dietary exposure: tracking movement and impacts in the gastrointestinal system. *Nanomaterials*, *5*(2), 1066-1086.

Martyniuk, C.J., Kroll, K.J., Doperalski, N.J., Barber, D.S. and Denslow, N.D., 2010. Environmentally relevant exposure to 17α-ethinylestradiol affects the telencephalic proteome of male fathead minnows. Aquatic toxicology, 98(4), pp.344-353.

Supplementary Table 2: SNEA illustrating regulation of cell processes in the gut

#	Total # of Neighbors	# of Measured Neighbors	Gene Set Seed	Median change	p-value
1	215	50	intestinal absorption	15.92	2.85E-07
2	122	17	gut development	31.84	1.19E-05
3	72	18	lipid absorption	47.80	5.68E-05
4	139	27	lipid export	19.38	2.99E-04
5	102	19	bile secretion	29.33	3.77E-04
6	155	18	lipoprotein metabolism	15.17	8.50E-04
7	44	10	gastrointestinal system absorption	106.84	8.68E-04
8	66	16	drug transport	29.33	1.33E-03
9	47	9	gastrointestinal system digestion	115.65	1.45E-03
10	573	47	energy homeostasis	6.83	1.71E-03
11	209	19	transcytosis	8.63	1.85E-03
12	100	15	intestine function	29.33	1.96E-03
13	245	22	fluid secretion	7.26	2.00E-03
14	159	22	intestine barrier	23.44	2.13E-03
15	55	13	gallstone formation	31.43	2.68E-03
16	99	10	tissue morphogenesis	18.22	3.36E-03
17	2433	201	vascularization	4.79	6.94E-03
18	130	9	gonad development	20.75	6.99E-03
19	389	38	lactation	10.19	7.03E-03
20	63	12	placenta transfer	13.97	7.38E-03
21	122	7	stem cell expansion	22.74	7.39E-03
22	70	10	intestine secretion	16.70	8.25E-03
23	449	58	thrombocyte aggregation	4.85	8.27E-03

24	118	12	postmenopause	10.58	8.37E-03
25	215	20	myelopoiesis	24.98	9.69E-03
26	249	21	decidualization	6.65	9.96E-03
27	96	5	gastric motility	115.65	1.03E-02
28	27	5	kidney elimination	23.44	1.03E-02
29	115	12	endothelialization	11.54	1.12E-02
30	109	10	pancreas development	31.84	1.15E-02
31	56	5	luteal maintenance	23.44	1.27E-02
32	79	9	gene duplication	18.25	1.33E-02
33	1332	133	pregnancy	4.74	1.38E-02
34	356	26	engraftment	7.58	1.51E-02
35	462	33	epithelial cell proliferation	6.41	1.60E-02
36	31	5	peptide transport	23.44	1.69E-02
37	56	7	desquamation	19.11	1.72E-02
38	133	20	drug metabolism	8.07	1.82E-02
39	120	14	epithelium development	43.89	1.82E-02
40	23	7	hepatobiliary excretion	23.44	1.84E-02
41	228	29	liver metabolism	7.98	1.86E-02
42	36	6	sugar metabolism	68.64	1.93E-02
43	62	5	fibroblast accumulation	14.91	2.01E-02
44	404	45	lipid transport	7.38	2.05E-02
45	57	5	stomach secretion	18.22	2.06E-02
46	517	34	reproduction	6.56	2.11E-02
47	354	26	receptor internalization	6.41	2.13E-02
48	153	12	neural crest cell development	16.08	2.18E-02
49	338	26	keratinocyte proliferation	7.62	2.26E-02
50	75	13	Pentose-phosphate shunt	29.33	2.56E-02
51	220	16	epithelial cell differentiation	18.25	2.57E-02
52	437	51	hemato-encephalic barrier	5.21	2.60E-02

53	361	18	regulation of action potential	5.94	2.64E-02
54	143	25	liver uptake	7.98	2.66E-02
55	166	17	hatching	6.26	2.70E-02
56	123	5	imprinting	9.20	3.02E-02
57	354	23	cell aggregation	6.98	3.02E-02
58	52	5	glycogenesis	115.65	3.11E-02
59	701	62	cell-cell adhesion	5.23	3.11E-02
60	127	7	endocrine function	18.22	3.38E-02
61	426	41	placenta development	6.65	3.40E-02
62	216	27	antigen binding	5.94	3.47E-02
63	51	6	vocalization	65.88	3.57E-02
64	96	5	sensory perception	59.08	3.58E-02
65	95	7	adrenal gland function	6.56	3.59E-02
66	155	23	renal reabsorption	7.09	3.73E-02
67	68	5	prenatal development	7.62	3.74E-02
68	303	19	cell communication	7.34	3.74E-02
69	100	7	penile erection	34.12	3.80E-02
70	57	10	hyperosmotic stress	11.45	3.83E-02
71	83	5	odontoblast differentiation	11.21	3.86E-02
72	264	27	acid secretion	4.85	3.93E-02
73	264	21	cardiogenesis	10.78	3.96E-02
74	28	5	mucosal immune response	47.93	4.02E-02
75	116	11	somitogenesis	10.78	4.26E-02
76	73	9	hibernation	10.30	4.64E-02

Supplementary Table 3: SNEA illustrating regulation of cell processes in the liver

#	Total # of Neighbors	# of Measured Neighbors	Gene Set Seed	Median change	p-value
1	174	18	fibrinolysis	81.23	6.46E-06
2	78	15	blood clot lysis	81.23	7.66E-06
3	242	13	neutrophil chemotaxis	215.03	3.36E-04
4	428	36	blood clotting	20.37	5.90E-04
5	449	39	thrombocyte aggregation	15.89	1.65E-03
6	158	16	microcirculation	25.18	3.01E-03
7	356	18	neutrophil recruitment	14.89	3.02E-03
8	81	8	sex maturation	90.17	3.17E-03
9	170	11	blood vessel contraction	19.58	4.39E-03
10	207	21	hemostasis	17.51	6.09E-03
11	308	18	neutrophil activation	10.36	1.04E-02
12	79	5	gene duplication	135.09	1.12E-02
13	330	26	liver development	17.51	1.13E-02
14	494	29	blood vessel permeability	16.88	1.14E-02
15	65	7	endothelial cell production	88.85	1.16E-02
16	62	11	anticoagulation	46.14	1.16E-02
17	120	11	epithelium development	22.09	1.36E-02
18	78	5	fibroblast function	25.18	1.39E-02
19	209	11	transcytosis	13.26	1.44E-02
20	91	10	Leukocyte-endothelial cell interaction	19.58	1.50E-02
21	438	40	hepatic regeneration	9.67	1.66E-02

22	409	23	tissue remodeling	13.26	1.72E-02
23	355	15	ECM degradation	13.26	1.92E-02
24	122	10	coronary artery blood flow	17.51	2.15E-02
25	484	27	kidney function	10.00	2.18E-02
26	1332	95	pregnancy	6.77	2.24E-02
27	74	10	vascular endothelium function	12.11	2.27E-02
28	108	7	muscle metabolism	30.71	2.51E-02
29	190	15	capillary permeability	13.26	2.57E-02
30	325	12	immunomodulation	47.50	2.81E-02
31	631	41	fertilization	8.51	3.11E-02
32	64	5	menopause	14.89	3.22E-02
33	129	11	leukocyte accumulation	10.00	3.41E-02
34	52	8	glycogenesis	70.15	3.45E-02
35	813	27	Ca++ export	12.11	3.70E-02
36	122	15	gut development	8.66	3.72E-02
37	106	10	Glycogen degradation	9.45	3.73E-02
38	492	10	microtubule cytoskeleton assembly	35.74	4.06E-02
39	320	24	endothelial cell adhesion	13.26	4.12E-02
40	191	19	hemolysis	12.11	4.24E-02
41	221	14	neutrophil adhesion	17.51	4.35E-02
42	171	9	thymus development	10.64	4.48E-02
43	42	6	lung blood flow	28.16	4.54E-02
44	228	31	liver metabolism	7.90	4.64E-02
45	816	45	cell damage	6.74	4.71E-02
46	72	11	lipid absorption	7.90	4.73E-02
47	164	7	menstrual cycle	9.67	4.91E-02
48	172	12	fetus growth	9.67	4.94E-02

Supplementary Table 4: SNEA illustrating regulation of cell processes in the telencephalon

#	Total # of Neighbors	# of Measured Neighbors	Gene Set Seed	Median change	p-value
1	264	10	neuron development	9.19	2.23E-03
2	105	7	forebrain development	6.26	3.31E-03
3	1129	21	neurogenesis	3.31	5.76E-03
4	182	7	cell fate specification	3.80	2.08E-02
5	425	9	axonogenesis	4.01	2.23E-02
6	2002	26	transcription activation	3.31	2.36E-02
7	492	9	stem cell proliferation	2.37	3.51E-02
8	136	5	neurulation	6.26	3.57E-02
9	478	6	organogenesis	4.30	3.68E-02
10	471	8	neuronal migration	5.87	4.04E-02
11	5848	63	cell differentiation	2.77	4.17E-02
12	346	7	axon guidance	3.38	4.23E-02
13	207	10	neuron differentiation	2.82	4.34E-02
14	6886	62	cell proliferation	2.61	4.83E-02
15	1107	23	cell fate	2.42	4.86E-02
16	446	6	neuronal plasticity	7.95	4.89E-02

Supplementary Table 5: SNEA illustrating regulation of cell processes in the hypothalamus

#	Total # of Neighbors	Overlap	Percent Overlap	Gene Set Seed	p-value
1	6991	39	0	cell differentiation	1.04E-05
2	109	5	4	nigrostriatal dopaminergic system	2.37E-05
3	62	4	6	pigment biosynthesis	4.43E-05
4	319	7	2	neuron development	6.03E-05
5	1100	12	1	brain development	1.31E-04
6	641	9	1	postnatal development	1.59E-04
7	3701	24	0	cell survival	1.83E-04
8	526	8	1	central nervous system development	2.22E-04
9	1017	11	1	nervous system development	2.78E-04
10	40	3	7	chondrocyte development	2.82E-04
11	41	3	7	intermediate filament polymerization	3.03E-04
12	1405	13	0	neurogenesis	3.36E-04
13	439	7	1	skeletal development	4.26E-04
14	1457	13	0	nerve cell differentiation	4.77E-04
15	338	6	1	axon cargo transport	6.46E-04
16	951	10	1	locomotion	6.76E-04
17	1142	11	0	ossification	7.38E-04
18	1146	11	0	adipogenesis	7.60E-04
19	1752	14	0	neurite outgrowth	8.41E-04
20	13	2	14	hypothalamus development	8.47E-04
21	361	6	1	chondrocyte differentiation	9.08E-04
22	138	4	2	gastric motility	9.28E-04
23	3877	23	0	cell migration	9.85E-04

24	8098	38	0	cell proliferation	1.01E-03
25	374	6	1	stem cell maintenance	1.09E-03
26	15	2	12	outflow tract morphogenesis	1.11E-03
27	251	5	1	trophoblast differentiation	1.13E-03
28	252	5	1	central nervous system function	1.15E-03
29	255	5	1	melanogenesis	1.21E-03
30	16	2	11	neuroimmunomodulation	1.26E-03
31	266	5	1	nerve development	1.45E-03
32	159	4	2	pituitary gland function	1.56E-03
33	18	2	10	joint function	1.58E-03
34	18	2	10	mesenchymal cell apoptosis	1.58E-03
35	887	9	1	transmission of nerve impulse	1.64E-03
36	19	2	10	olfactory bulb development	1.75E-03
37	430	6	1	nerve regeneration	2.21E-03
38	87	3	3	physiological stress	2.61E-03
39	960	9	0	neurotransmission	2.81E-03
40	1156	10	0	heart function	2.91E-03
41	25	2	7	adrenal gland development	2.95E-03
42	91	3	3	reinnervation	2.97E-03
43	969	9	0	developmental process	2.99E-03
44	26	2	7	aorta flow	3.18E-03
45	1800	13	0	regeneration	3.27E-03
46	805	8	0	osteoblast differentiation	3.43E-03
47	471	6	1	cell aggregation	3.46E-03
48	28	2	6	glial cell development	3.67E-03
49	1616	12	0	memory	3.83E-03
50	29	2	6	glycerol biosynthesis from pyruvate	3.92E-03
51	30	2	6	blood-aqueous barrier	4.18E-03
52	106	3	2	nerve potential	4.53E-03

53	347	5	1	mesenchymal stem cell differentiation	4.55E-03
54	665	7	1	cognition	4.55E-03
55	215	4	1	intestine motility	4.60E-03
56	1660	12	0	cell development	4.75E-03
57	217	4	1	dopaminergic system	4.75E-03
58	1448	11	0	neuronal death	4.80E-03
59	4900	25	0	cell death	4.82E-03
60	353	5	1	appetite	4.89E-03
61	110	3	2	Schwann cell migration	5.02E-03
62	2358	15	0	oxidative stress	5.12E-03
63	224	4	1	heat-shock response	5.31E-03
64	114	3	2	immobilization stress	5.54E-03
65	35	2	5	response to pain	5.61E-03
66	35	2	5	pineal gland function	5.61E-03
67	36	2	5	adrenal secretion	5.92E-03
68	372	5	1	cardiovascular system development	6.08E-03
69	233	4	1	limb development	6.09E-03
70	376	5	1	ontogeny	6.35E-03
71	121	3	2	hypothalamus function	6.53E-03
72	38	2	5	limb regeneration	6.56E-03
73	1515	11	0	aging	6.71E-03
74	382	5	1	long-term memory	6.78E-03
75	123	3	2	striatal dopamine release	6.82E-03
76	1105	9	0	lumen formation	7.01E-03
77	243	4	1	neuron differentiation	7.05E-03
78	388	5	1	fluid secretion	7.23E-03
79	40	2	4	operant conditioning	7.23E-03
80	40	2	4	adrenocortical secretion	7.23E-03
81	247	4	1	brain blood flow	7.46E-03

82	42	2	4	homophilic cell adhesion	7.93E-03
83	42	2	4	endothelium development	7.93E-03
84	5086	25	0	cell growth	7.98E-03
85	400	5	1	cell fate determination	8.19E-03
86	133	3	2	adrenal gland function	8.44E-03
87	944	8	0	neuroprotection	8.77E-03
88	135	3	2	micturition	8.79E-03
89	410	5	1	muscle contraction	9.05E-03
90	270	4	1	osteocyte function	1.01E-02
91	425	5	1	innervation	1.05E-02
92	1393	10	0	cell fate	1.06E-02
93	49	2	4	hippocampus development	1.06E-02
94	49	2	4	neurotransmitter uptake	1.06E-02
95	50	2	3	hormone biosynthesis	1.10E-02
96	50	2	3	stomach function	1.10E-02
97	2338	14	0	DNA replication	1.16E-02
98	52	2	3	vestibule function	1.19E-02
99	612	6	0	neuronal migration	1.19E-02
100	153	3	1	uterus contractility	1.23E-02

Supplementary Table 6: Expression Targets Derived from Pathway Studio for Gut, Liver and Brain

Name	Description
ABCA1	ATP-binding cassette, sub-family A (ABC1), member 1
ABCB11	ATP-binding cassette, sub-family B (MDR/TAP), member 11
ADCYAP1	adenylate cyclase activating polypeptide 1 (pituitary)
ADCYAP1R1	adenylate cyclase activating polypeptide 1 (pituitary) receptor type I
AKT2	v-akt murine thymoma viral oncogene homolog 2
ARAF	v-raf murine sarcoma 3611 viral oncogene homolog
ASCL1	achaete-scute complex homolog 1 (Drosophila)
ATXN7	ataxin 7
AXL	AXL receptor tyrosine kinase
BDNF	brain-derived neurotrophic factor
BDNF-AS	BDNF antisense RNA
BMP2	bone morphogenetic protein 2
BMP4	bone morphogenetic protein 4
BMPR1A	bone morphogenetic protein receptor, type IA
C1D	C1D nuclear receptor corepressor
C1QTNF1	C1q and tumor necrosis factor related protein 1
CDX1	caudal type homeobox 1
CDX2	caudal type homeobox 2
CEBPA	CCAAT/enhancer binding protein (C/EBP), alpha
CEBPB	CCAAT/enhancer binding protein (C/EBP), beta
CHRM3	cholinergic receptor, muscarinic 3
CLOCK	clock homolog (mouse)
CNTF	ciliary neurotrophic factor
CREB1	cAMP responsive element binding protein 1

CRHR2	corticotropin releasing hormone receptor 2
CXCR4	chemokine (C-X-C motif) receptor 4
CYP19A1	cytochrome P450, family 19, subfamily A, polypeptide 1
CYP27B1	cytochrome P450, family 27, subfamily B, polypeptide 1
DDC	dopa decarboxylase (aromatic L-amino acid decarboxylase)
DDIT3	DNA-damage-inducible transcript 3
DICER1	dicer 1, ribonuclease type III
DKK1	dickkopf 1 homolog (Xenopus laevis)
DRD2	dopamine receptor D2
EGR1	early growth response 1
ESR1	estrogen receptor 1
ESRRG	estrogen-related receptor gamma
ETV5	ets variant 5
F7	coagulation factor VII (serum prothrombin conversion accelerator)
FGF13	fibroblast growth factor 13
FGF19	fibroblast growth factor 19
FGF8	fibroblast growth factor 8 (androgen-induced)
FGFR1	fibroblast growth factor receptor 1
FGFR2	fibroblast growth factor receptor 2
FGFR4	fibroblast growth factor receptor 4
FOXA1	forkhead box A1
FOXA2	forkhead box A2
FOXA3	forkhead box A3
FOXM1	forkhead box M1
FOXN4	forkhead box N4
FOXO1	forkhead box O1
FRMD7	FERM domain containing 7
FST	follistatin
G6PC	glucose-6-phosphatase, catalytic subunit

GATA2	GATA binding protein 2
GATA4	GATA binding protein 4
GATA5	GATA binding protein 5
GATA6	GATA binding protein 6
GDF2	growth differentiation factor 2
GDNF	glial cell derived neurotrophic factor
GFPT1	glutamine--fructose-6-phosphate transaminase 1
GFRA1	GDNF family receptor alpha 1
GHRH	growth hormone releasing hormone
GHRL	ghrelin/obestatin prepropeptide
GNRHR	gonadotropin-releasing hormone receptor
GRHL2	grainyhead-like 2 (Drosophila)
GSC	goosecoid homeobox
HNF1A	HNF1 homeobox A
HNF1B	HNF1 homeobox B
HNF4A	hepatocyte nuclear factor 4, alpha
HNF4G	hepatocyte nuclear factor 4, gamma
HOXA1	homeobox A1
HRAS	v-Ha-ras Harvey rat sarcoma viral oncogene homolog
HTR2A	5-hydroxytryptamine (serotonin) receptor 2A, G protein-coupled
HTR2C	5-hydroxytryptamine (serotonin) receptor 2C, G protein-coupled
IGF1	insulin-like growth factor 1 (somatomedin C)
IL1A	interleukin 1, alpha
IL1B	interleukin 1, beta
IL6	interleukin 6 (interferon, beta 2)
INS	insulin
IRS1	insulin receptor substrate 1
KL	klotho
LDLR	low density lipoprotein receptor

LEF1	lymphoid enhancer-binding factor 1
LEP	leptin
LIFR	leukemia inhibitory factor receptor alpha
LMX1A	LIM homeobox transcription factor 1, alpha
m_Ggta1	glycoprotein, alpha-galactosyltransferase 1,3
m_Olfr54	olfactory receptor 54
m_Penk-rs	preproenkephalin, related sequence
m_Scpro2	stem cell proliferation 2
MAFB	v-maf musculoaponeurotic fibrosarcoma oncogene homolog B (avian)
MAPK3	mitogen-activated protein kinase 3
MECP2	methyl CpG binding protein 2 (Rett syndrome)
MIR23A	microRNA 23a
MIR34A	microRNA 34a
MMP13	matrix metallopeptidase 13 (collagenase 3)
MYOG	myogenin (myogenic factor 4)
NEFM	neurofilament, medium polypeptide
NEUROD1	neuronal differentiation 1
NEUROG1	neurogenin 1
NEUROG2	neurogenin 2
NEUROG3	neurogenin 3
NF1	neurofibromin 1
NFE2	nuclear factor, erythroid 2
NGF	nerve growth factor (beta polypeptide)
NKX3-1	NK3 homeobox 1
NOG	noggin
NPRL3	nitrogen permease regulator-like 3 (S. cerevisiae)
NPY1R	neuropeptide Y receptor Y1
NR0B1	nuclear receptor subfamily 0, group B, member 1
NR0B2	nuclear receptor subfamily 0, group B, member 2

NR1H3	nuclear receptor subfamily 1, group H, member 3
NR1H4	nuclear receptor subfamily 1, group H, member 4
NR1I2	nuclear receptor subfamily 1, group I, member 2
NR1I3	nuclear receptor subfamily 1, group I, member 3
NR2F2	nuclear receptor subfamily 2, group F, member 2
NR4A2	nuclear receptor subfamily 4, group A, member 2
NR5A2	nuclear receptor subfamily 5, group A, member 2
NTF3	neurotrophin 3
NTF4	neurotrophin 4
NTRK1	neurotrophic tyrosine kinase, receptor, type 1
NTRK2	neurotrophic tyrosine kinase, receptor, type 2
OLIG1	oligodendrocyte transcription factor 1
OLIG2	oligodendrocyte lineage transcription factor 2
ONECUT1	one cut homeobox 1
OTP	orthopedia homeobox
OXT	oxytocin/neurophysin I prepropeptide
PAX7	paired box 7
PBX1	pre-B-cell leukemia homeobox 1
PCSK1	proprotein convertase subtilisin/kexin type 1
PCSK2	proprotein convertase subtilisin/kexin type 2
PCSK9	proprotein convertase subtilisin/kexin type 9
PHOX2B	paired-like homeobox 2b
PITX3	paired-like homeodomain 3
PKNOX1	PBX/knotted 1 homeobox 1
POU4F1	POU class 4 homeobox 1
PPARA	peroxisome proliferator-activated receptor alpha
PPARGC1B	peroxisome proliferator-activated receptor gamma, coactivator 1 beta
PREP	prolyl endopeptidase
PRKAA1	protein kinase, AMP-activated, alpha 1 catalytic subunit

PRKACA	protein kinase, cAMP-dependent, catalytic, alpha
PRLH	prolactin releasing hormone
PRLR	prolactin receptor
PRMT1	protein arginine methyltransferase 1
PROC	protein C (inactivator of coagulation factors Va and VIIIa)
PROS1	protein S (alpha)
PRR14	proline rich 14
PRRX1	paired related homeobox 1
PYY	peptide YY
QRSL1	glutaminyl-tRNA synthase (glutamine-hydrolyzing)-like 1
QSOX1	quiescin Q6 sulfhydryl oxidase 1
r_Chga	chromogranin A (parathyroid secretory protein 1)
RAMP1	receptor (G protein-coupled) activity modifying protein 1
REST	RE1-silencing transcription factor
RNANC	Retinal nonattachment, nonsyndromic congenital
RUNX1T1	runt-related transcription factor 1; translocated to, 1 (cyclin D-related)
RXRA	retinoid X receptor, alpha
SALL3	sal-like 3 (Drosophila)
SCD	stearoyl-CoA desaturase (delta-9-desaturase)
SHH	sonic hedgehog
SIN3A	hypothetical protein LOC795324
SLC2A1	solute carrier family 2 (facilitated glucose transporter), member 1
SMAD2	SMAD family member 2
SMAD3	SMAD family member 3
SMAD4	SMAD family member 4
SOX3	SRY (sex determining region Y)-box 3
SOX8	SRY (sex determining region Y)-box 8
SOX9	SRY (sex determining region Y)-box 9
SREBF1	sterol regulatory element binding transcription factor 1

SYP	synaptophysin
TCF3	transcription factor 3 (E2A immunoglobulin enhancer binding factors E12/E47)
TCF7L2	transcription factor 7-like 2 (T-cell specific, HMG-box)
TFAP2D	transcription factor AP-2 delta (activating enhancer binding protein 2 delta)
TGFB1	transforming growth factor, beta 1
TNF	tumor necrosis factor
TOM1L2	target of myb1-like 2 (chicken)
TRH	thyrotropin-releasing hormone
TRIB3	tribbles homolog 3 (Drosophila)
UCN	urocortin
USF1	upstream transcription factor 1
VEGFA	vascular endothelial growth factor A
WNT1	wingless-type MMTV integration site family, member 1
WNT2	wingless-type MMTV integration site family member 2
WNT3A	wingless-type MMTV integration site family, member 3A
YWHAE	tyrosine 3-monooxygenase/tryptophan 5-monooxygenase activation protein, epsilon polypeptide
YY1	YY1 transcription factor
ZEB1	zinc finger E-box binding homeobox 1

Supplementary Table 7. Subnetwork enrichment analysis for gut based on proteomics data

#	Total # of Neighbors	# of Measured Neighbors	Gene Set Seed	Median change	p-value
1	159	5	intestine barrier	4.13	4.41E-04
2	1250	42	cytoskeleton organization and biogenesis	2.45	4.59E-04
3	1343	51	contraction	2.37	3.59E-03
4	129	8	actin filament depolymerization	3.46	4.19E-03
5	2433	67	vascularization	2.45	5.26E-03
6	1662	41	cell invasion	2.37	6.42E-03
7	1368	56	actin organization	2.34	6.87E-03
8	124	6	tissue invasion	4.35	8.81E-03
9	701	19	cell-cell adhesion	2.38	1.07E-02
10	1715	54	cell motility	2.37	1.26E-02
11	306	14	focal adhesion assembly	2.80	1.53E-02
12	95	11	actin filament bundle assembly	3.42	1.78E-02
13	115	5	transmembrane signaling	3.10	1.82E-02
14	266	5	eye morphogenesis	3.10	2.08E-02
15	96	6	muscle fiber contraction	3.41	2.09E-02
16	72	5	lipid absorption	3.20	2.13E-02
17	289	9	membrane ruffling	2.67	2.14E-02
18	1141	28	wound healing	2.39	2.14E-02
19	80	8	virus assemblies	3.20	2.34E-02
20	238	9	smooth muscle contraction	2.52	2.37E-02
21	378	8	establishment of cell polarity	2.87	2.67E-02
22	80	6	adherens junction assembly	2.88	2.78E-02
23	100	5	intestine function	4.13	2.81E-02
24	418	14	cell contact	2.38	2.90E-02
25	323	6	innervation	4.02	3.02E-02
26	186	5	pattern specification	3.55	3.09E-02
27	354	10	cell aggregation	2.57	3.39E-02

28	108	9	diastolic function	2.91	3.57E-02
29	5848	136	cell differentiation	2.20	3.72E-02
30	144	6	cell blebbing	2.38	3.93E-02
31	56	9	Fatty acids import	2.76	4.33E-02
32	630	24	regulation of cell shape	2.37	4.51E-02
33	264	6	cardiogenesis	2.57	4.55E-02
34	256	5	mammary gland development	3.95	4.57E-02
35	155	9	lipoprotein metabolism	2.76	4.64E-02
36	1854	56	inflammatory response	2.32	4.72E-02
37	150	6	smooth muscle cell differentiation	2.80	4.86E-02

Supplementary Table 8. Subnetwork enrichment analysis for liver based on proteomics data

#	Total # of Neighbors	# of Measured Neighbors	Gene Set Seed	Median change	p-value
1	472	53	detoxification	3.37	6.77E-05
2	153	27	xenobiotic clearance	3.55	7.56E-05
3	523	31	gluconeogenesis	3.42	3.78E-04
4	228	17	liver metabolism	3.58	1.06E-03
5	80	13	nucleotide biosynthesis	3.85	3.09E-03
6	370	7	acidification	3.75	4.49E-03
7	1002	63	lipid metabolism	2.97	5.20E-03
8	341	22	liver function	3.42	7.17E-03
9	330	9	liver development	3.60	7.81E-03
10	37	5	anaerobic glycolysis	3.85	9.74E-03
11	105	9	nitrosative stress	3.36	1.22E-02
12	362	9	lymphocyte proliferation	3.42	1.26E-02
13	15	6	transsulfuration	4.17	1.41E-02
14	216	16	kidney excretion	3.42	1.53E-02
15	699	48	Respiratory chain	3.01	1.58E-02
16	284	9	cell fusion	3.42	1.68E-02
17	497	16	DNA methylation	3.36	2.00E-02
18	72	5	sperm capacitation	4.04	2.09E-02
19	890	18	T lymphocyte proliferation	3.33	2.14E-02
20	378	7	cytokine response	3.84	2.23E-02
21	63	8	ketone biosynthesis	3.10	2.28E-02
22	487	19	brain function	3.37	2.35E-02
23	438	16	hepatic regeneration	3.16	3.23E-02
24	292	7	S-G2 transition	3.85	3.43E-02
25	1793	102	oxidative stress	2.73	3.44E-02
26	469	30	glycolysis	2.98	3.48E-02

27	228	7	hepatic stellate cell activation	3.42	3.50E-02
28	59	9	protein unfolding	3.02	3.51E-02
29	235	9	oogenesis	3.13	3.62E-02
30	484	13	kidney function	3.58	4.01E-02
31	456	12	macrophage activation	3.33	4.04E-02
32	558	11	DNA strand breakage	3.37	4.23E-02
33	806	34	glucose import	2.67	4.38E-02
34	341	18	response to oxidative stress	3.37	4.48E-02
35	124	8	salinity response	3.53	4.49E-02
36	361	6	regulation of action potential	3.57	4.54E-02
37	430	18	NO biosynthesis	3.56	4.74E-02

Supplementary Table 9. Subnetwork enrichment analysis for brain based on proteomics data

#	Total # of Neighbors	Overlap	Percent Overlap	Gene Set Seed	p-value
1	2358	39	1	oxidative stress	4.56E-10
2	1752	32	1	neurite outgrowth	2.56E-09
3	40	7	17	neurotransmitter secretion	3.31E-09
4	132	10	7	synaptic vesicle transport	4.99E-09
5	1416	28	1	life span	5.89E-09
6	1720	31	1	actin organization	6.68E-09
7	8098	77	0	cell proliferation	1.52E-08
8	934	22	2	immunoreactivity	1.59E-08
9	3701	47	1	cell survival	2.41E-08
10	1613	29	1	cytoskeleton organization and biogenesis	2.47E-08
11	984	22	2	Respiratory chain	4.02E-08
12	1457	27	1	nerve cell differentiation	4.53E-08
13	1800	30	1	regeneration	7.26E-08
14	7686	72	0	apoptosis	1.95E-07
15	4900	53	1	cell death	4.47E-07
16	904	19	2	cell spreading	9.70E-07
17	998	20	2	long-term synaptic potentiation	1.01E-06
18	92	7	7	protein unfolding	1.09E-06
19	1017	20	1	synaptogenesis	1.36E-06
20	1515	25	1	aging	1.37E-06
21	1994	29	1	endocytosis	2.14E-06
22	319	11	3	non-selective vesicle fusion	2.42E-06
23	68	6	8	synaptic vesicle endocytosis	2.83E-06

24	1386	23	1	exocytosis	3.57E-06
25	1616	25	1	memory	4.34E-06
26	3877	43	1	cell migration	5.69E-06
27	118	7	5	mitochondrial transport	5.70E-06
28	1128	20	1	synaptic transmission	6.48E-06
29	754	16	2	learning and/or memory	6.77E-06
30	361	11	3	vesicle-mediated transport	7.84E-06
31	951	18	1	locomotion	8.26E-06
32	960	18	1	neurotransmission	9.39E-06
33	301	10	3	acrosome reaction	9.83E-06
34	525	13	2	membrane fusion	1.06E-05
35	130	7	5	vesicle docking	1.07E-05
36	1703	25	1	pregnancy	1.08E-05
37	609	14	2	neuronal plasticity	1.10E-05
38	1936	27	1	internalization	1.14E-05
39	529	13	2	protein folding	1.15E-05
40	612	14	2	neuronal migration	1.16E-05
41	312	10	3	protein aggregation	1.34E-05
42	54	5	9	non-selective vesicle targeting	1.59E-05
43	195	8	4	xenobiotic clearance	1.79E-05
44	257	9	3	Tricarboxylic acid cycle	1.85E-05
45	556	13	2	spindle assembly	1.94E-05
46	5086	50	0	cell growth	2.13E-05
47	1124	19	1	synaptic plasticity	2.21E-05
48	59	5	8	neuromuscular function	2.44E-05
49	338	10	2	axon cargo transport	2.66E-05
50	429	11	2	visual learning	3.85E-05
51	782	15	1	mitochondrial depolarization	4.32E-05
52	115	6	5	synaptic vesicle exocytosis	5.62E-05

53	1100	18	1	brain development	5.68E-05
54	6991	61	0	cell differentiation	6.23E-05
55	73	5	6	synaptic vesicle fusion	6.76E-05
56	549	12	2	lipid peroxidation	7.86E-05
57	550	12	2	cell contact	8.00E-05
58	841	15	1	regulation of cell shape	9.77E-05
59	41	4	9	intermediate filament polymerization	9.80E-05
60	319	9	2	hemolysis	9.81E-05
61	1952	25	1	protein synthesis	1.04E-04
62	42	4	9	homophilic cell adhesion	1.08E-04
63	663	13	1	cell motion	1.17E-04
64	955	16	1	membrane depolarization	1.17E-04
65	2723	31	1	immune response	1.18E-04
66	84	5	5	retrograde protein transport ER to cytosol	1.31E-04
67	1985	25	1	cell invasion	1.36E-04
68	136	6	4	endocytic recycling	1.41E-04
69	1651	22	1	proteolysis	1.67E-04
70	689	13	1	detoxification	1.70E-04
71	1210	18	1	growth rate	1.89E-04
72	1448	20	1	neuronal death	2.16E-04
73	526	11	2	central nervous system development	2.32E-04
74	1029	16	1	heart development	2.72E-04
75	2235	26	1	mitosis	3.45E-04
76	647	12	1	breathing	3.58E-04
77	1059	16	1	T-cell response	3.74E-04
78	957	15	1	drug resistance	3.94E-04
79	854	14	1	virulence	3.99E-04
80	473	10	2	oxidative phosphorylation	4.13E-04
81	63	4	6	free radical scavenging	5.05E-04

82	673	12	1	brain function	5.08E-04
83	113	5	4	sperm capacitation	5.13E-04
84	116	5	4	mitochondrion fusion	5.78E-04
85	1447	19	1	viral reproduction	5.90E-04
86	409	9	2	interphase	6.09E-04
87	181	6	3	cell redox homeostasis	6.48E-04
88	29	3	10	protein thiol-disulfide exchange	6.70E-04
89	599	11	1	stress fiber assembly	6.88E-04
90	122	5	4	Glut/Gln/Pro metabolsm	7.25E-04
91	30	3	9	Golgi reassembly	7.39E-04
92	705	12	1	cell polarity	7.63E-04
93	3042	31	1	vascularization	8.40E-04
94	127	5	3	autolysis	8.68E-04
95	265	7	2	ADCC	8.79E-04
96	620	11	1	myelination	9.12E-04
97	1151	16	1	cell damage	9.17E-04
98	838	13	1	DNA fragmentation	1.08E-03
99	275	7	2	neural crest cell migration	1.09E-03
100	134	5	3	actin filament bundle assembly	1.10E-03
